# Automated Installation Checking and Hanger Pre-Adjustment for Stone Curtain Walls Based on UAV Photogrammetry and Terrestrial Laser Scanning

**DOI:** 10.3390/s26206647

**Published:** 2026-10-21

**Authors:** Pengteng Lin, Mayong Cai, Dejun Mo, Xiaoming Luo, Yingjie Wang, Hongzhi Cui, Jinxiang Li, Jiyuan Chen, Shuai Wen, Yi Tan

**Affiliations:** 1 China Construction Fifth Division South China Company, Guangzhou 510220, Chinawyj1240679488@163.com (Y.W.);; 2 Sino-Australia Joint Research Center in BIM and Smart Construction, Shenzhen University, Shenzhen 518060, China; 3 College of Civil and Transportation Engineering, Shenzhen University, Shenzhen 518060, China

**Keywords:** dry-hanging stone curtain wall, fused point cloud, hanger pre-adjustment, installation checking, multi-point attachment

## Abstract

The installation quality of multi-point attachment unit systems in stone curtain walls depends on the geometric compatibility between unit-side hanger arrays and support-side attachment nodes (SSANs). However, deviations from on-site welding, adapter component installation, and end-connection positioning can accumulate within individual SSANs, affecting hanger seating, synchronized multi-point attachment, and joint-width coordination. Therefore, this study proposes an installation checking and hanger pre-adjustment method based on UAV photogrammetry and terrestrial laser scanning (TLS). First, UAV close-range photogrammetry and TLS are used to acquire complementary point clouds of the installed SSANs and the surrounding curtain wall inspection area. Second, SSANs and their internal components are extracted, and end-connection positioning features are determined using a Point–Line–Plane geometric framework. Third, the measured geometric relationships among SSAN positioning features are converted into target hanger layout parameters to assess installation compatibility and generate hanger pre-adjustment and installation decisions. Finally, the proposed method was evaluated in a real project. The average total installation time per curtain wall unit was 48 min compared with 70 min for the traditional method, while the average number of on-site adjustments per curtain wall unit was 4 compared with 15 for the traditional method. The joint-width range was 18.6–23.1 mm compared with 16.8–27.4 mm. The field comparison showed lower average installation time and adjustment counts and a narrower joint-width range in the proposed-method group.

## 1. Introduction

**Figure 1:**
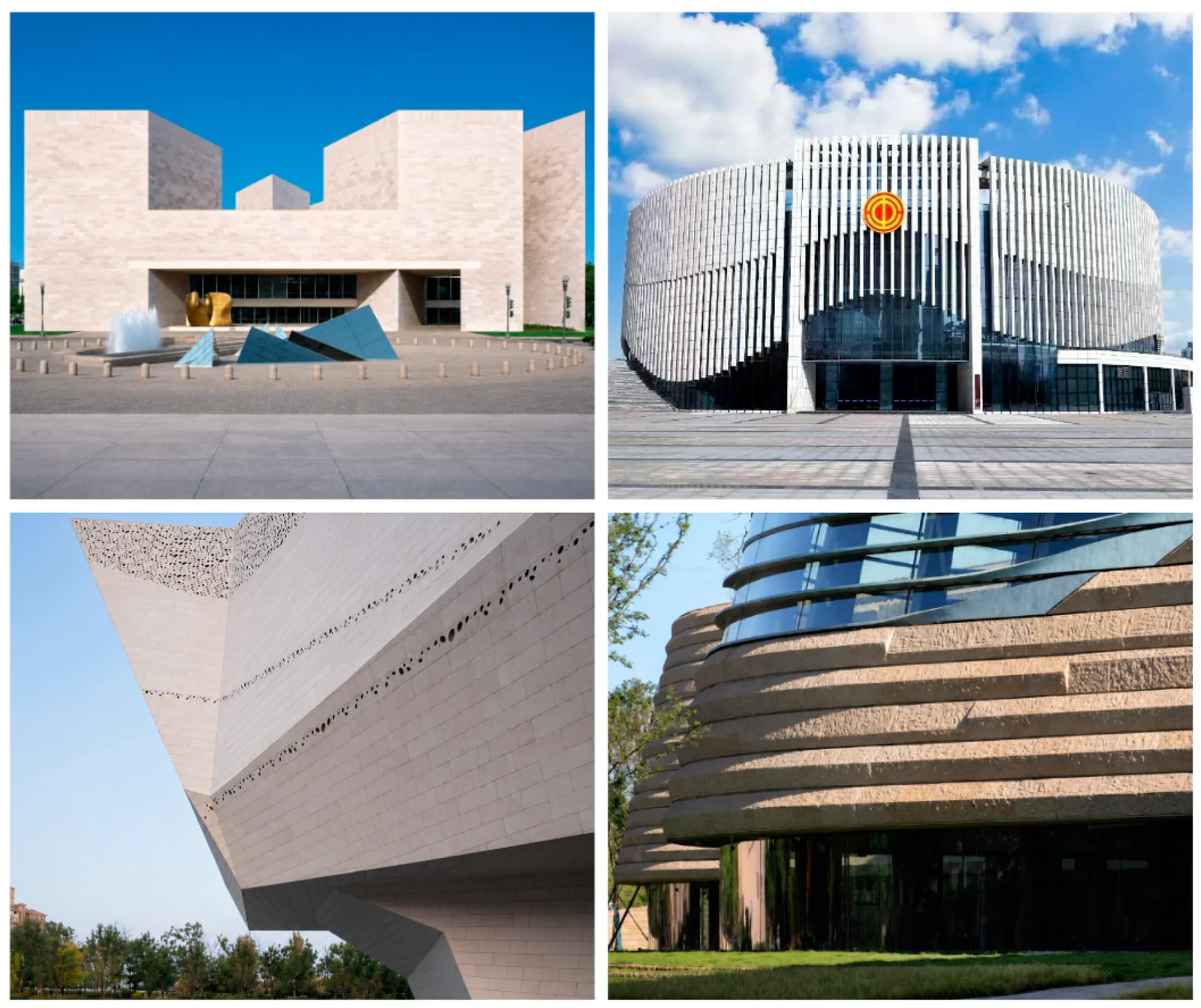
Representative applications and diverse facade forms of stone curtain walls.

Stone curtain walls are widely used in public buildings, commercial complexes, cultural and sports venues, and landmark buildings because of their material texture, durability, and facade expression. Representative applications and diverse facade forms of stone curtain walls are shown in Figure 1. With the development of complex curved facades, irregular panel divisions, and prefabricated construction, unitized configurations with multi-point attachment features have been increasingly adopted in dry-hanging stone curtain walls. These configurations can accommodate variations in wall curvature, panel division direction, and installation sequence [1,2]. Such systems usually consist of stone panels, sub-frames, unit-side hangers, and support-side attachment nodes (SSANs). Their installation is characterized by coordinated multi-point attachment between multiple unit-side hangers within one curtain wall unit and multiple SSANs.

In actual construction, SSANs usually consist of projecting support members, adapter components, and end-connection components. These components are installed through welding, bolted connections, and on-site adjustment. Deviations in the main structure, support-member welding, adapter-component installation, and end-connection positioning are transferred progressively within each SSAN and are ultimately reflected in its end-connection positioning feature. For a multi-point attachment unit system, the installation compatibility of a curtain wall unit is governed by the overall geometric relationship of the corresponding SSAN group. Satisfactory positioning of an individual SSAN does not necessarily ensure synchronized seating of multiple attachment points. Coupled deviations among SSAN spacing, unit-side hanger seating positions, and curtain wall design boundaries may prevent individual attachment points from seating properly, exceed the available hanger adjustment range, require repeated trial installation, or impair joint-width coordination.

Traditional total station layout, string-line checking, and manual measurement can provide control points, elevations, and local dimensional information. However, these methods cannot efficiently represent the internal component states of SSANs, their end-connection positioning features, or the relative geometric relationships within an SSAN group. In traditional installation, the compatibility between an SSAN group and the corresponding unit-side hanger array mainly relies on on-site trial installation. A mismatch between the measured relative relationships within the SSAN group and the adjustable range of the hangers can lead to repeated high-altitude trial installation, detachment and reattachment, and manual adjustment. This increases construction time, site coordination cost, and operational risk. Therefore, pre-lifting installation checking of multi-point attachment unit systems should focus on the compatibility between the measured relative relationships of SSAN groups and the adjustable range of unit-side hangers. A conversion method is also required to transform the measured support-side geometric state into hanger pre-adjustment parameters and installation decisions.

Existing studies have advanced curtain wall construction quality control from the perspectives of construction measurement, post-installation quality inspection, safety inspection of existing facades, and curtain wall installation automation. Three-dimensional laser scanning and BIM have been used for curtain wall construction error analysis, detailed design feedback, and installation plan correction [3]. Non-contact sensing technologies, such as colored point clouds, images, and infrared data, have also been applied to defect detection in dry-hanging stone curtain walls and safety inspection of existing facades [4,5]. Studies on curtain wall installation automation mainly focus on component transportation, lifting, pose control, and installation execution [6,7,8]. These studies provide an important foundation for measured deviation identification, post-installation quality evaluation, and installation execution. However, their outputs are mainly geometric deviations, quality evaluation results, or installation execution information. For multi-point attachment curtain wall systems, such information does not directly answer whether the measured geometric configuration of an SSAN group can be accommodated by the corresponding unit-side hanger array before lifting, nor does it provide the hanger adjustment values and support-side rechecking information required for installation.

Therefore, the key issue addressed in this study is the conversion from measured support-side geometry to actionable pre-lifting installation parameters and decisions, rather than point-cloud-based dimensional inspection itself. To address this issue, this study proposes an installation-checking and hanger pre-adjustment method for SSANs in a multi-point attachment unit system of dry-hanging stone curtain walls. UAV photogrammetry and terrestrial laser scanning are used as complementary sensing techniques to acquire the as-built geometric information required for the proposed workflow. The method targets SSANs that have already been installed on the building facade before curtain wall unit lifting and converts the measured relative geometric relationships among SSANs into unit-side hanger pre-adjustment parameters and installation decisions. Specifically, discrete SSANs are organized through spatial logical indexing, while representative points, layout baselines, and feature planes are used to characterize the positions, arrangement relationships, and orientations of their internal components. End-connection positioning features are then extracted as geometric intermediates linking the support-side SSAN groups with the corresponding unit-side hanger arrays. Based on these features, the measured SSAN relationships are converted into target hanger layout distances and boundary positioning distances and are compared with the available hanger adjustment ranges. Relationships that cannot be accommodated through hanger adjustment are further linked to the corresponding SSAN indices and component-level checking results to guide support-side rechecking and adjustment. In this way, the proposed method extends point-cloud-based geometric measurement from deviation identification to hanger pre-adjustment and pre-lifting installation decision-making.

The main contributions of this study are as follows:

(1) A multi-point attachment compatibility conversion method is established to transform measured SSAN geometric relationships into actionable hanger pre-adjustment parameters. The measured relationships among end-connection positioning features are converted into target hanger layout distances and boundary positioning distances, thereby bridging the gap between support-side as-built geometric measurement and unit-side hanger pre-adjustment.
(2) A pre-lifting installation-decision and deviation-tracing mechanism is developed. By comparing the converted target relationships with the available hanger adjustment ranges, the method identifies relationships that can be accommodated through hanger pre-adjustment and those requiring support-side rechecking. The latter are further linked to the corresponding SSAN indices and component-level checking results, providing explicit information for pre-lifting installation decisions.
(3) A component-level geometric checking framework is developed to support the above conversion and decision-making process. Spatial logical indexing and point-, line-, and plane-based geometric relationships are used to characterize the measured positions, layout baselines, and orientations of internal SSAN components and to extract the end-connection positioning features required for multi-point attachment compatibility assessment.

The remainder of this paper is organized as follows. Section 2 reviews installation measurement and quality inspection of dry-hanging stone curtain walls, multi-source point cloud fusion modeling, and the application of point cloud-based measured geometry in component installation checking. Section 3 presents the proposed multi-source point cloud-driven method for SSAN checking and hanger pre-adjustment. Section 4 validates the feasibility and performance of the proposed method through an engineering case. Section 5 summarizes the conclusions and discusses future research directions.

## 2. Literature Review

### 2.1. Installation Measurement and Quality Inspection of Curtain Walls

The installation quality of dry-hanging stone curtain walls is jointly affected by deviations in the main structure, positioning deviations in the support system, installation errors of internal components of SSANs, and on-site adjustment. For the multi-point attachment unit system investigated in this study, the unit-side hanger array must form a multi-point correspondence with the installed SSANs. Therefore, installation checking should not only consider the positional state of an individual SSAN, but also evaluate the measured relative geometric relationships among SSANs and their geometric compatibility with the unit-side hanger array.

Studies on curtain wall installation automation have emphasized positioning accuracy, pose control, and stable alignment during component lifting. Yu et al. [6] developed and tested a curtain wall installation robot. Johns et al. [7] reviewed automation technologies for curtain wall installation in high-rise buildings. Iturralde et al. [8] proposed a cable-driven parallel robot for curtain wall module installation. These studies mainly focus on component transportation, pose control, and installation execution. Their target installation positions or connection interfaces are generally treated as predefined inputs. Consequently, limited attention has been paid to the pre-lifting compatibility between the measured geometric configuration of SSANs and unit-side hanger arrays.

Non-contact sensing and digital measurement technologies have been widely applied to curtain wall inspection and construction feedback. Yao et al. [4] used colored point clouds for automated defect detection in dry-hanging stone curtain walls, while Chew and Gan [5] reviewed automated inspection and maintenance methods for potentially falling facade components. Wang et al. [3] further combined three-dimensional laser scanning and BIM for curtain wall construction error analysis, fabrication dimension correction, and installation plan adjustment. These studies demonstrate the value of measured data for defect identification and deviation feedback, but they mainly address post-installation inspection or measured-to-design comparison rather than pre-lifting attachment compatibility.

In summary, existing studies have supported curtain wall installation execution, defect inspection, and construction deviation analysis. However, the measured geometry of SSANs has not been sufficiently converted into hanger pre-adjustment parameters and installation decisions. Therefore, a pre-lifting method is still needed to assess multi-point attachment compatibility, identify out-of-range relationships, and reduce repeated adjustments at height.

### 2.2. Multi-Source Point Cloud Fusion in Measured Construction Scene Modeling

Terrestrial laser scanning offers good scale stability and spatial measurement capability [9] and can acquire dense three-dimensional coordinate data. However, data acquisition in elevated areas, on the rear sides of components, and in occluded regions is often constrained by scanner placement and line-of-sight conditions. UAV photogrammetry and close-range photogrammetry provide flexible viewpoints and rich texture information [10], thereby compensating for incomplete observations of complex facades and local details. Consequently, multi-source point cloud fusion can provide complementary coverage and improve the completeness of measured construction scene models.

Pu and Vosselman [11] integrated terrestrial laser scanning point clouds with image information for building facade reconstruction. Their results showed that the combined use of geometric and texture information improved facade representation. Teza et al. [12] compared three-dimensional laser scanning with structure-from-motion photogrammetry for architectural morphology analysis and identified differences in accuracy, completeness, and operational efficiency between the two data sources. Luhmann et al. [13] integrated UAV photogrammetry, ground-based photography, and laser scanning data for the three-dimensional reconstruction of historic buildings. Their study demonstrated that combining aerial and ground-based data improved the geometric and textural representation of complex buildings.

For multi-platform point cloud registration, Park et al. [14] proposed an automated framework for registering UAV and UGV point clouds using local image features. The framework first estimates an initial transformation from corresponding image feature points and then applies iterative closest point refinement to integrate point clouds acquired from different platforms. Their study demonstrates that aerial and ground-based datasets can be registered within a common spatial reference, thereby complementing spatial coverage and reducing local blind spots. Bouziani et al. [15] evaluated building models generated using UAV photogrammetry and terrestrial laser scanning. Their results demonstrated the complementary performance of UAV and TLS data in roof and elevated areas, facade detail capture, and scale stability. Mohammadi et al. [16] assessed digital twin models generated from UAV photogrammetry and TLS data in a bridge case study. They found that point density, occlusion, registration, and surface type affected the accuracy and completeness of the fused models. Together, these studies show that multi-source point cloud fusion can compensate for the limitations of a single measurement method in spatial coverage, local detail, and scale stability, thereby providing more complete measured geometric data for complex construction scenes.

In summary, existing studies have demonstrated that multi-source point cloud fusion can improve spatial coverage, local detail, and registration consistency in complex construction scenes. However, its application to pre-lifting curtain wall installation checking remains limited, particularly in SSAN extraction, end-connection positioning feature identification, and hanger pre-adjustment parameter generation. Therefore, multi-source fused point clouds are introduced in this study to provide the measured geometric basis for multi-point attachment compatibility assessment and hanger pre-adjustment.

### 2.3. Point Cloud-Based Measured Geometry in Component Installation Checking

Point-cloud-based measured geometry must be converted into indicators for construction quality inspection and installation checking through component recognition, feature extraction, and spatial relationship analysis. Bosché [17] proposed a method for automatically recognizing 3D CAD model objects in laser-scanned point clouds and calculating measured dimensions, thereby providing a basis for point-cloud-based dimensional compliance checking. Son et al. [18] combined color information with three-dimensional data for structural component recognition and modeling. Maalek et al. [19] automatically recognized common structural components in reinforced concrete construction from point clouds and used the recognition results for progress monitoring and dimensional quality control. These studies demonstrate that point cloud data can be converted into computable indicators of component position, dimensions, shape, and spatial relationships.

In prefabricated and modular construction, point clouds have been widely used to inspect component dimensions, surface quality, and installation state. Kim et al. [20] established a framework combining BIM and three-dimensional laser scanning for the dimensional and surface-quality assessment of precast concrete components. Tan et al. [21] used BIM and LiDAR for automated geometric quality inspection of prefabricated housing units. Tan et al. [22] further applied BIM and LiDAR to automated geometric quality inspection of modular boxes. Guo et al. [23] proposed a BIM- and LiDAR-based method for automated geometric quality inspection of precast utility tunnel components. Wang et al. [24] used laser scanning for on-site installation quality inspection of prefabricated wall panels. These studies demonstrate that point-cloud-based measured geometry can support the evaluation of dimensional deviations, surface quality, position, orientation, and installation status across different component types.

Beyond single-component quality inspection, point-cloud-based measured geometry has also been used for assembly tolerance and interface compatibility control. Li et al. [25] combined three-dimensional laser scanning and BIM to improve assembly tolerance control in a modular demountable floodwall project. Kalasapudi and Tang [26] conducted automated tolerance analysis of curved prefabricated components using three-dimensional point clouds. Long et al. [27] analyzed installation tolerances in prefabricated construction using dimensional chain theory and noted that final interface deviations may result from cumulative errors in fabrication, positioning, and assembly. Tran et al. [28] and Iturralde et al. [29] linked measured point clouds, module layouts, fabrication information, and on-site assembly in studies of prefabricated facade digital twins and renovation workflows. These studies indicate that point clouds can support not only component geometric quality inspection but also assembly compatibility analysis through interface relationships, relative positions, and error propagation.

In summary, point-cloud-based measured geometry has been widely used for component recognition, dimensional inspection, installation-state evaluation, and assembly tolerance analysis. However, existing studies provide limited support for coordinated multi-point attachment, particularly for analyzing relative relationships among multiple components and mapping observable geometric features to concealed attachment conditions. Therefore, a configuration-specific method is still required for pre-lifting compatibility checking, hanger pre-adjustment, and installation decision-making.

## 3. Methodology

**Figure 2:**
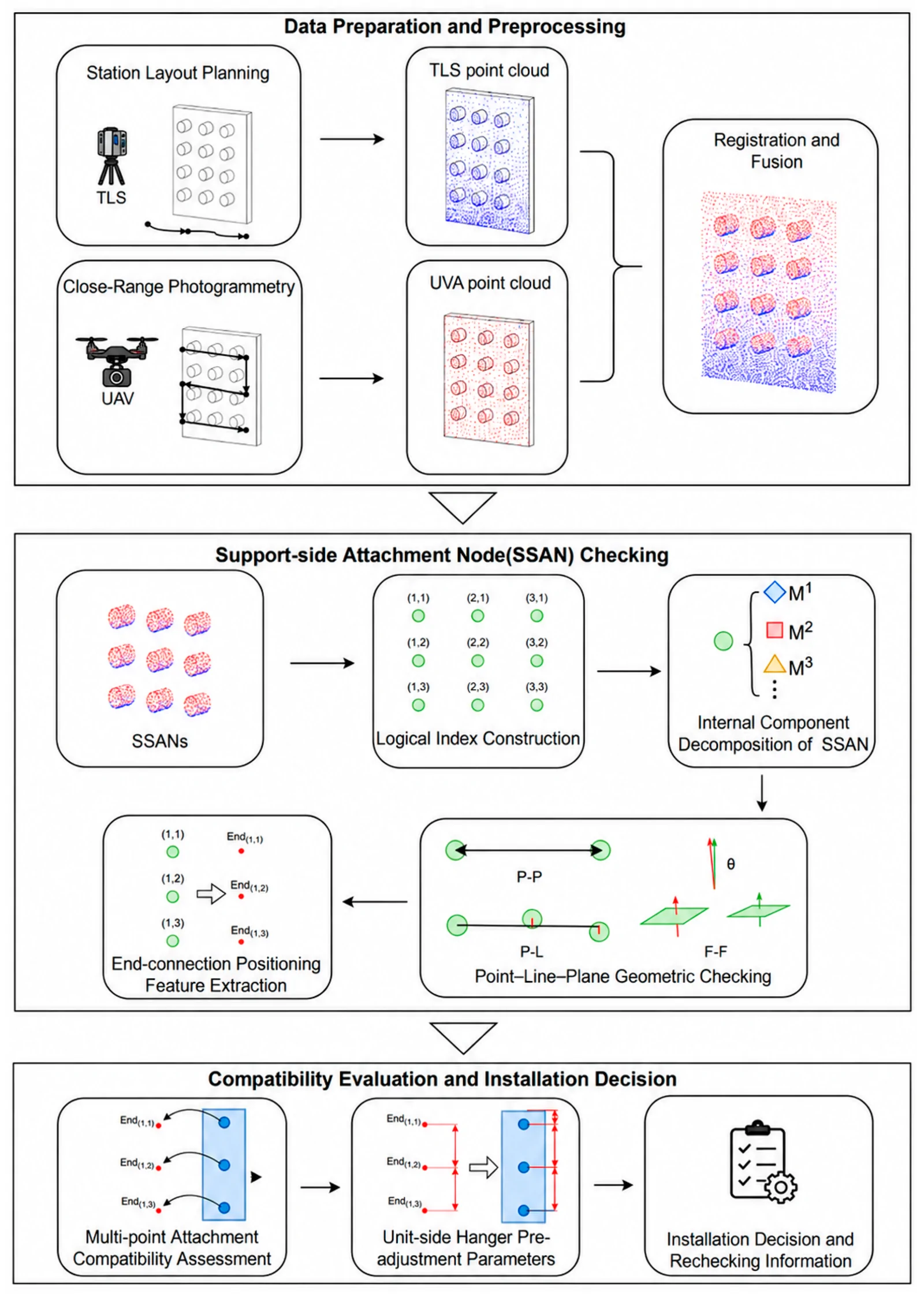
Technical workflow of the proposed method.

This study proposes an SSAN checking and curtain wall unit hanger pre-adjustment method based on UAV photogrammetry and TLS point clouds. First, UAV close-range photogrammetry and TLS data are acquired and fused to reconstruct the curtain wall inspection area. Second, SSANs and their internal components are extracted, and the end-connection positioning features are determined using the Point–Line–Plane framework. Third, the measured geometric relationships are converted into target hanger layout parameters for installation compatibility assessment and hanger pre-adjustment decision-making. The workflow of the proposed method is shown in Figure 2.

### 3.1. Data Acquisition and 3D Reconstruction of Curtain Wall Inspection Area

SSAN checking requires both the overall layout relationships within SSAN groups and the local geometric details of their end-connection positioning features. A single data source cannot simultaneously provide adequate spatial coverage, scale accuracy, and local visibility. Therefore, this study combines scene-level environmental acquisition with detailed acquisition in the curtain wall inspection area. First, UAV oblique photogrammetry is used to reconstruct a general digital model of the construction scene. This model supports identification of the curtain wall inspection area, TLS station-layout conditions, and local occlusion relationships. It is then used to plan TLS stations and UAV close-range photogrammetry flight paths. High-accuracy TLS point clouds and multi-view close-range images of SSANs and surrounding components are subsequently acquired. Finally, the TLS and UAV close-range photogrammetry data are preprocessed, registered, and fused to generate a unified 3D point cloud model of the curtain wall inspection area, which provides the spatial-data basis for subsequent SSAN extraction and geometric checking.

#### 3.1.1. 3D Model Reconstruction for General Installation Scene

The curtain wall installation scene is reconstructed from UAV oblique photogrammetry data. Structure from Motion (SfM) and Multi-View Stereo (MVS) are used for feature matching, camera pose estimation, and dense reconstruction of multi-view images, thereby generating a three-dimensional construction scene model with spatial position information.

During data acquisition, a UAV equipped with a Real-Time Kinematic (RTK) positioning module is used to collect large-scale images of the inspection area. Corresponding Position and Orientation System (POS) data are recorded simultaneously. After data acquisition, three-dimensional reconstruction software is used to generate a real-scene digital model within a global coordinate reference. The model represents the building outline, variations in facade geometry, and the spatial distribution of construction-site obstacles. It also provides the basis for subsequent TLS station planning and UAV close-range photogrammetry path planning.

#### 3.1.2. TLS Station Planning and Data Acquisition

**Figure 3:**
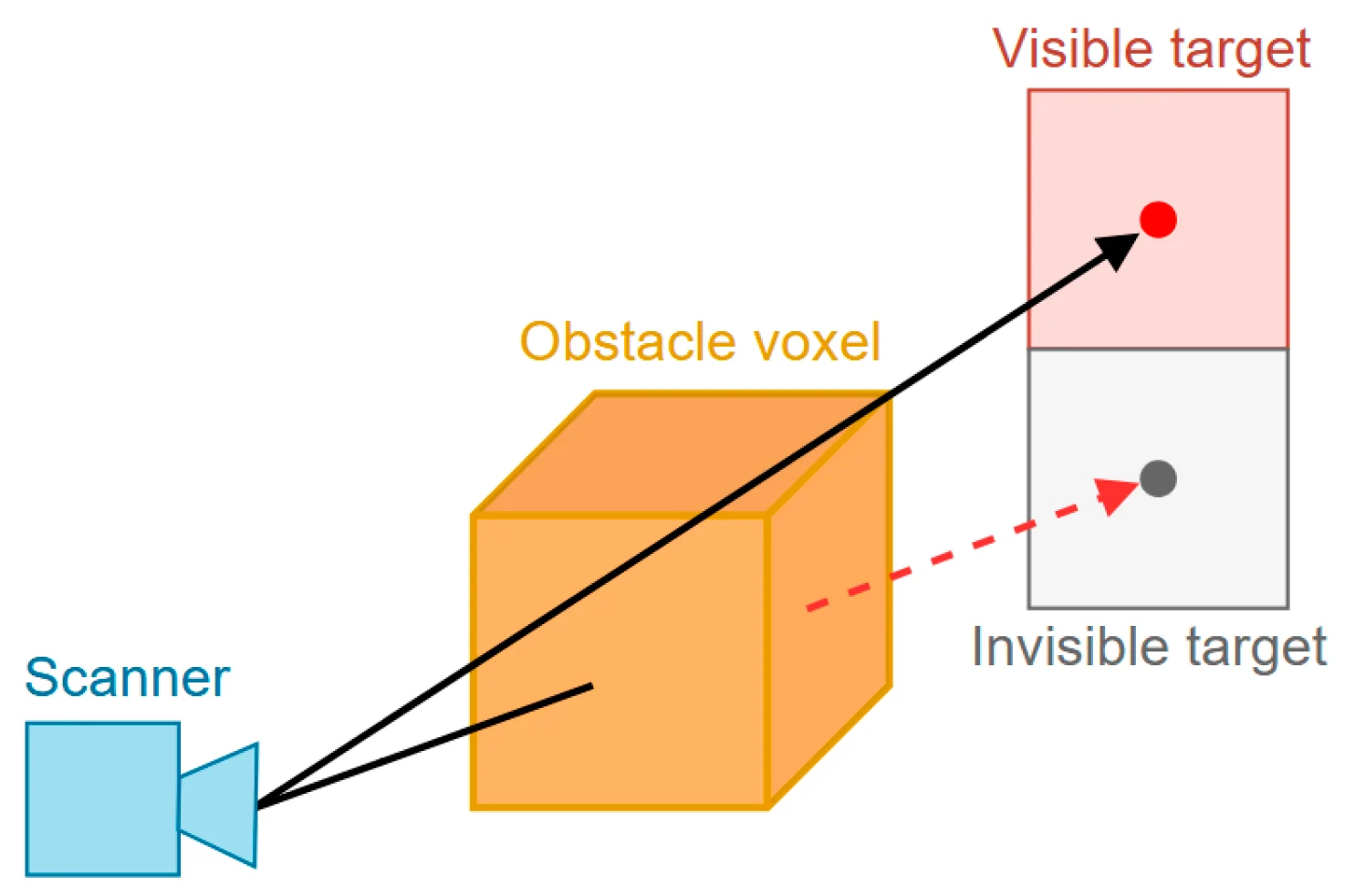
Visibility analysis for TLS station layout planning.

TLS point clouds provide the primary quantitative data for geometric checking of SSANs. Station planning is based on the environmental model. The curtain wall inspection area is first identified, and ground surfaces, floor slabs, or working platforms that satisfy scanner setup requirements are extracted as candidate station areas. Visibility between candidate stations and the curtain wall inspection area is then analyzed according to the scanner field of view, effective scanning distance, laser incidence angle, and on-site occlusion conditions, as shown in Figure 3.

After candidate stations are generated, a coverage-priority selection strategy is applied. Stations with high coverage of the curtain wall inspection area and sufficient mutual overlap are retained, whereas stations with limited marginal coverage or high redundancy are removed. The final station configuration should satisfy the requirements for SSAN visibility, point cloud completeness, inter-station registration stability, and on-site feasibility. After acquisition, point clouds from multiple stations are registered and merged using control points or geometric features in overlapping areas, thereby generating a TLS point cloud that covers the curtain wall inspection area.

#### 3.1.3. UAV Photogrammetry Data Acquisition and 3D Reconstruction

For SSANs at height, ground-based TLS can generally capture their outer and lower surfaces. However, blind spots may remain on upper surfaces, rear sides, and recessed areas. In addition, SSANs located far from the scanner may have relatively low point densities, which can affect component extraction and installation checking. UAV close-range photogrammetry is therefore used to supplement local geometric details of SSANs within the curtain wall inspection area. Compared with scene-level oblique photogrammetry, close-range photogrammetry focuses on continuous coverage, detailed texture, and local geometric representation of SSANs and adjacent components. Before path planning, the boundary of the curtain wall inspection area is extracted from the environmental model. Scene data unrelated to the checking task are removed to generate a planning model for viewpoint generation and flight-safety assessment. The acquisition parameters are determined according to the geometric representation requirements of SSANs and the identification requirements of end-connection positioning features.

Viewpoint generation is constrained by the target Ground Sampling Distance (GSD), camera field of view, image overlap, shooting distance, and flight safety distance. GSD represents the actual physical size on the curtain wall inspection surface corresponding to one image pixel. To ensure that SSANs are represented with sufficient geometric detail for subsequent feature extraction and analysis, the target resolution and image overlap are determined according to the requirements for identifying end-connection positioning features.

**Figure 4:**
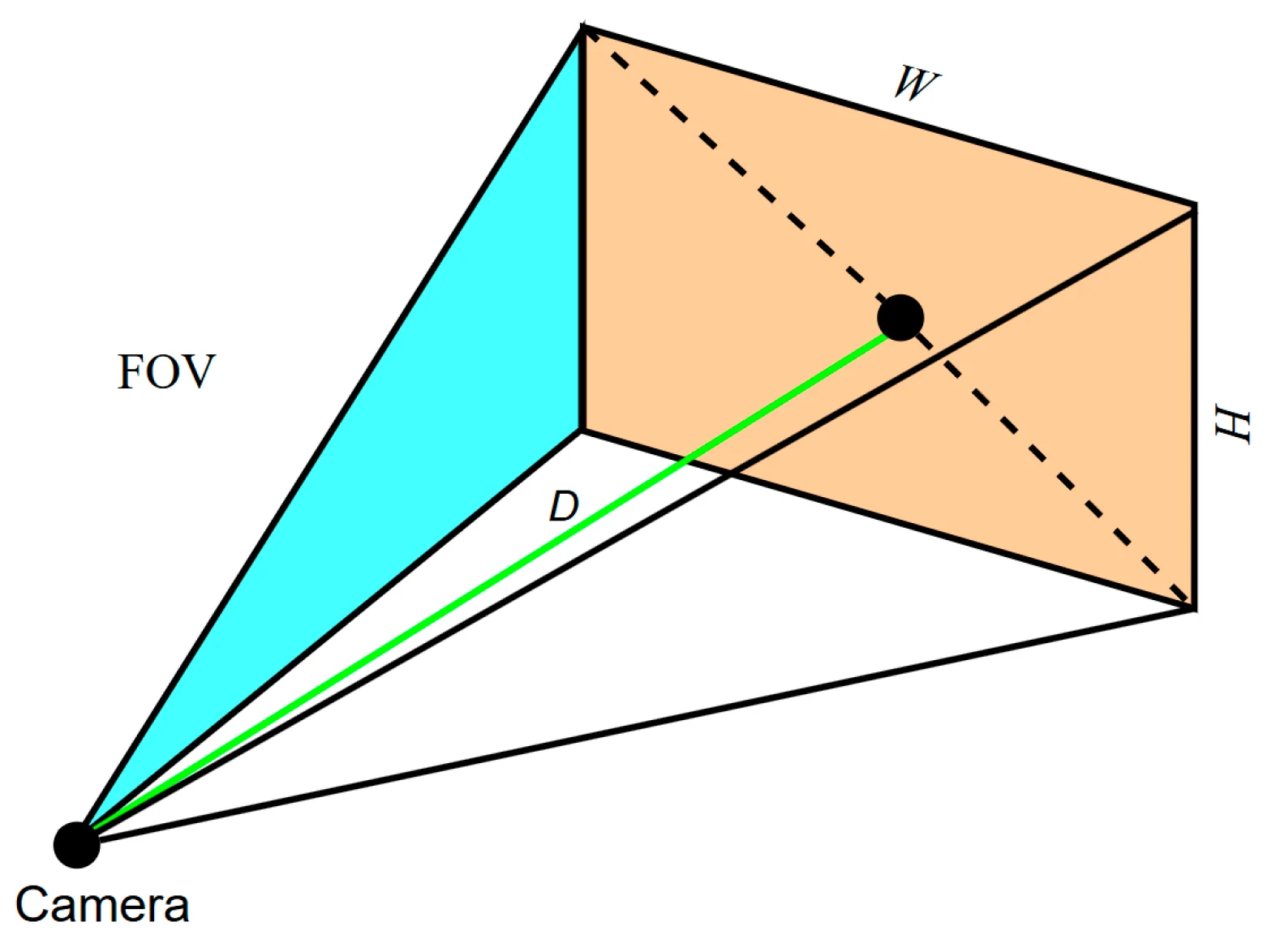
Relationship between camera field of view and shooting distance. The cyan region illustrates the camera field of view, while the orange plane represents the target surface.

The target GSD is first determined according to the required geometric resolution of end-connection positioning features. The perpendicular shooting distance *D* from the camera center to the target surface is then calculated from the camera focal length and physical pixel size, as shown in Equation (1). After *D* is determined, the horizontal and vertical coverage of a single image on the target facade is calculated from the horizontal and vertical fields of view, as shown in Equation (2):

(1) $$\mathrm{GSD}=\frac{D\times a}{f}$$

(2) $$\{\begin{array}{c} W=2D\tan\left(\frac{\mathrm{FO}V_{h}}{2}\right)\\ H=2D\tan\left(\frac{\mathrm{FO}V_{v}}{2}\right) \end{array}$$

where *D* is the perpendicular distance from the camera center to the target surface, *a* is the physical size of a single sensor pixel, and *f* is the camera focal length. *W* and *H* are the horizontal and vertical coverage of a single image on the target surface, respectively. *FOV_h_* and *FOV_v_* are the horizontal and vertical camera fields of view, respectively. The relationship between camera field of view and shooting distance is illustrated in Figure 4.

**Figure 5:**
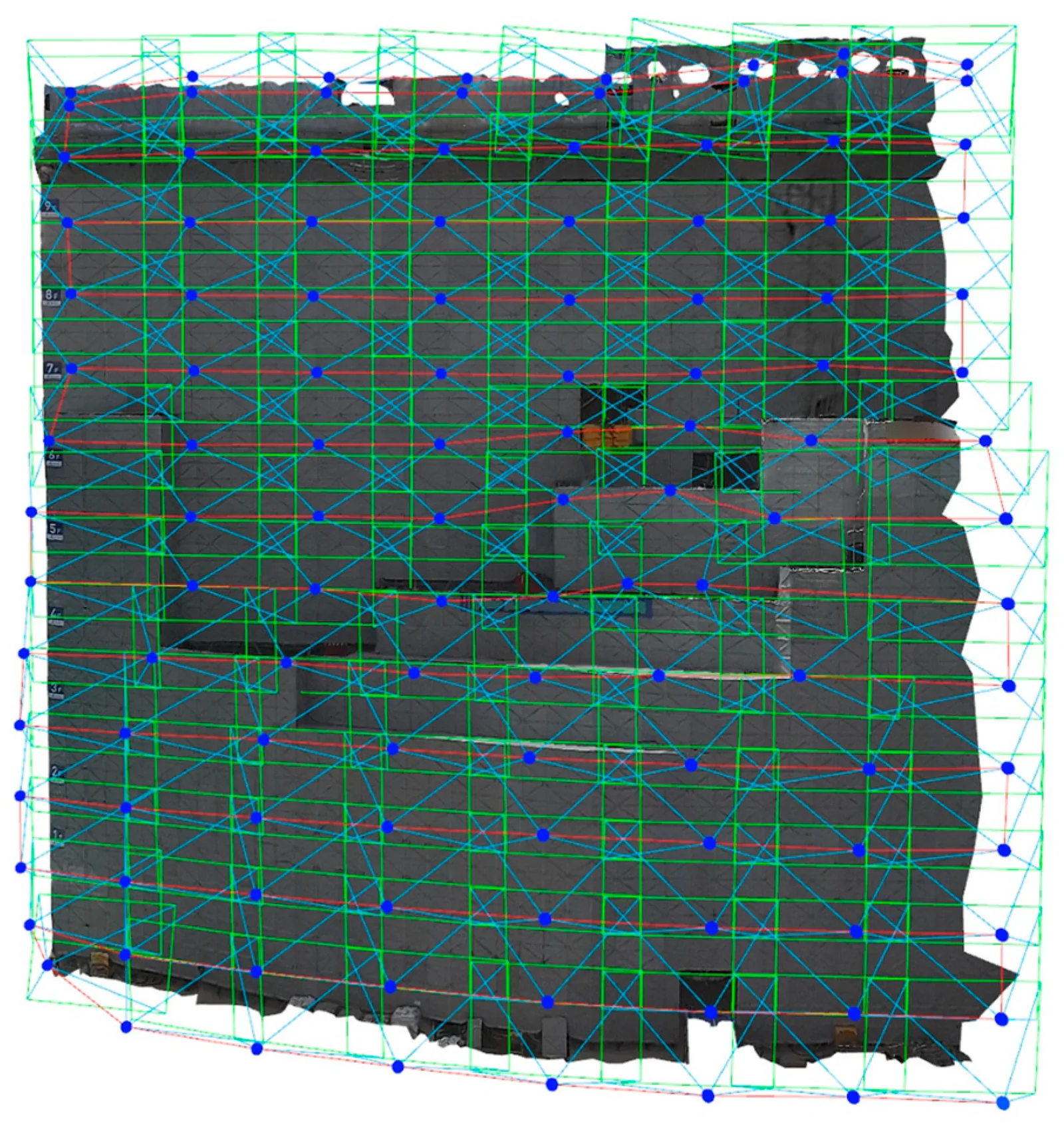
UAV close-range photogrammetry viewpoints and flight path.

The spacing and number of adjacent viewpoints are calculated from the shooting distance *D*, single-image coverage, forward overlap, and side overlap. A continuous close-range photogrammetry flight path covering the curtain wall inspection area is then generated according to spatial adjacency. The environmental model is subsequently used for safety-distance and collision checking. Local flight segments that conflict with site obstacles or safety constraints are removed, adjusted, or rerouted. Where UAV coverage is restricted by safety distance, occlusion, or flight conditions, manual close-range photography or other supplementary acquisition methods may be used. The verified flight path is used for close-range photogrammetry data acquisition in the curtain wall inspection area. The planned viewpoints and flight path are shown in Figure 5.

After data acquisition, close-range images are screened for quality. Blurred images, images with abnormal exposure, and images with insufficient overlap are removed. Photogrammetry software is then used for image matching, sparse point cloud generation, dense point cloud reconstruction, and texture mapping, thereby producing a local three-dimensional model of the curtain wall inspection area. This model supplements the visibility and local geometric completeness of SSAN regions and is subsequently integrated with the TLS point cloud through multi-source registration and fusion.

After scene-level and close-range data acquisition, the TLS point cloud and UAV close-range photogrammetry point cloud differ in coordinate reference, point density, coverage, and local error distribution. Preprocessing, registration, and fusion are therefore performed as the final stage of three-dimensional reconstruction to generate a unified spatial dataset. The TLS point cloud is used as the coordinate and scale reference, whereas the UAV close-range photogrammetry point cloud supplements local geometric details. The resulting fused point cloud model covers the curtain wall inspection area and provides the direct input for support-side SSAN extraction.

#### 3.1.4. Multi-Source Point Cloud Data Preprocessing

Raw TLS point clouds from multiple stations are merged and transformed into a unified coordinate system to form a TLS point cloud covering the curtain wall inspection area. The point cloud is then cropped according to the curtain wall unit range, field measurement extent, or construction zone, retaining only data relevant to SSAN checking. Statistical filtering and moderate downsampling are subsequently applied within the cropped area. Statistical filtering removes outliers and random noise, whereas downsampling reduces data redundancy and improves subsequent processing efficiency. The main geometric features of support members, adapter components, and end-connection interfaces must be preserved during preprocessing. Excessive simplification should be avoided because it may remove boundary points, positioning surfaces, or local attachment features. The processed TLS point cloud is used as the spatial reference for multi-source registration and fusion.

The UAV close-range photogrammetry point cloud is also inspected for quality. Regions containing obvious reconstruction errors caused by insufficient texture, reflection, occlusion, or reconstruction failure are removed. The point cloud is then locally cropped according to the curtain wall inspection area, retaining only valid data required for registration with the TLS point cloud and geometric supplementation of the SSANs.

#### 3.1.5. Multi-Source Point Cloud Registration and Fusion

The TLS point cloud was used as the reference dataset, and the UAV close-range photogrammetry point cloud was used as the source dataset. Before registration, the two datasets were manually cropped according to the curtain wall inspection area to remove irrelevant background data and retain the common overlapping region. The subsequent point-cloud registration was performed using the Point Cloud Library (PCL). For feature-based initial registration, component-related geometric regions were first extracted from both datasets. Intrinsic Shape Signatures (ISS) keypoints were detected, and Fast Point Feature Histograms (FPFH) were calculated to describe their local geometric characteristics. Candidate feature correspondences were automatically established according to descriptor similarity and spatial constraints, without manual point-pair selection. RANSAC-based correspondence rejection was then applied to remove geometrically inconsistent matches and estimate the feature-based transformation.

The transformation was subsequently refined using a multi-scale Generalized Iterative Closest Point (GICP) procedure with progressively reduced correspondence-distance thresholds, followed by point-to-plane ICP refinement using surface-normal information. After convergence, the UAV point cloud was transformed into the TLS coordinate system and fused with the TLS dataset.

**Figure 6:**
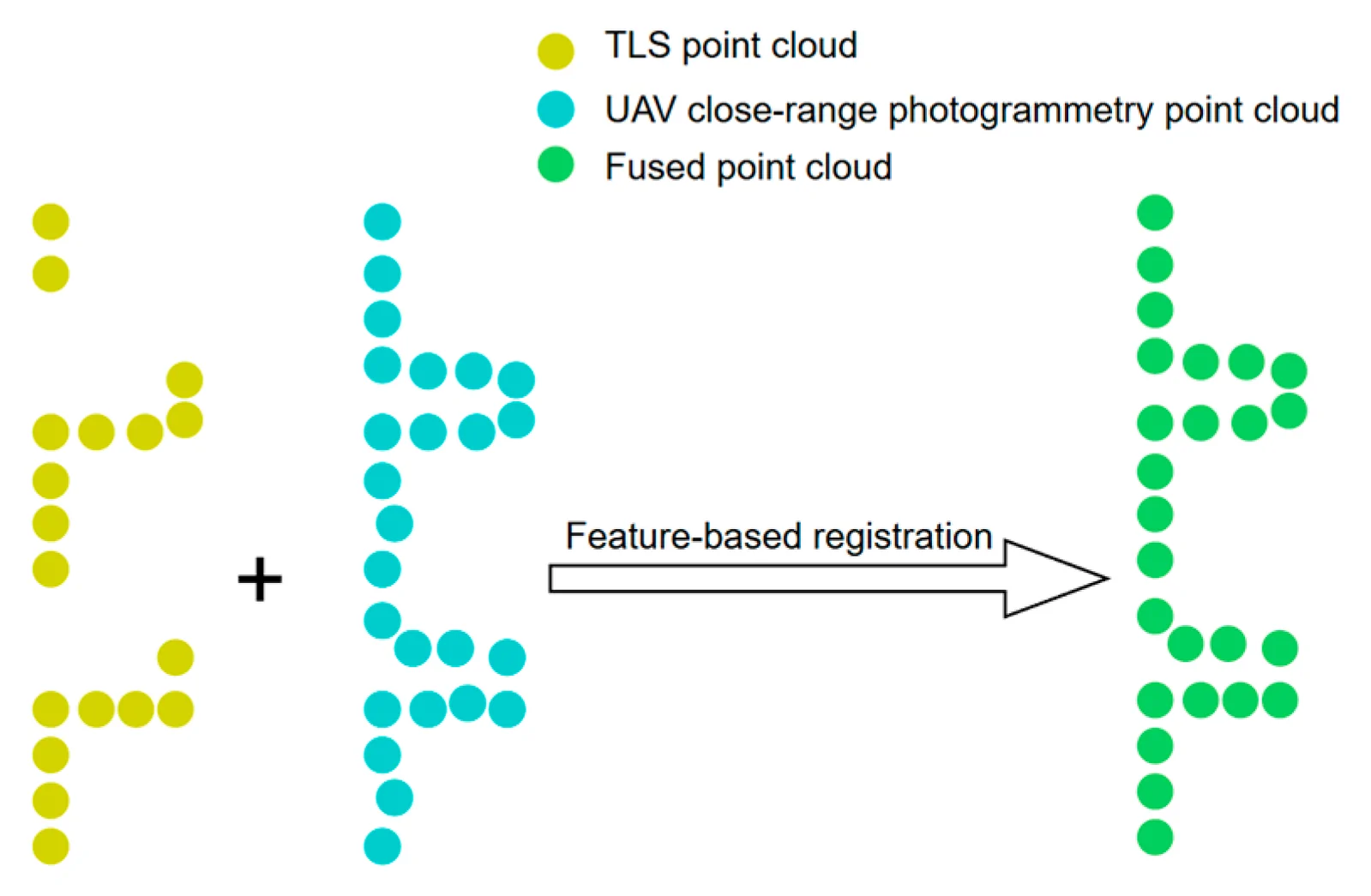
Registration and fusion of multi-source point clouds.

Registration quality was quantitatively evaluated using point-to-point and point-to-plane residuals between geometrically compatible point pairs in the overlapping component regions of the registered TLS and UAV datasets. In the fused point-cloud model, the TLS point cloud served as the primary metric reference for subsequent SSAN geometric checking and hanger pre-adjustment calculations, whereas the UAV point cloud supplemented geometric information from elevated, side, and locally occluded regions that were insufficiently observed by ground-based scanning. The multi-source point-cloud registration and fusion process is illustrated in Figure 6.

### 3.2. SSAN Checking and Positioning Feature Extraction

An SSAN generally consists of a support member, an adapter component, and an end-connection component. Installation deviations can propagate through these internal components and are ultimately reflected in the end-connection positioning feature. Consequently, extracting SSAN point-cloud clusters alone is insufficient for multi-point attachment compatibility assessment. The installation states of same-type components in different SSANs must also be checked. Accordingly, this section extracts individual SSANs, establishes their spatial indices and internal-component representations, constructs component-level geometric checking relationships using representative points, layout baselines, and feature planes, and extracts the end-connection positioning features that directly engage with unit-side hangers.

#### 3.2.1. SSAN Extraction

The fused point cloud model generated in Section 3.1 contains SSANs together with the substrate wall surface, temporary facilities, and other non-target data. To obtain individual SSAN point clouds for subsequent geometric checking, the substrate surface is first separated from the curtain wall inspection area, after which the discretely distributed protruding attachment structures are extracted.

First, the substrate wall surface or primary background surface within the curtain wall inspection area is fitted and separated. For regions with gradual curvature variation that satisfy the planar-approximation condition, Random Sample Consensus (RANSAC) is used to identify the background plane and calculate its normal vector. If the substrate surface is curved or the local planar-fitting residual exceeds a specified threshold, a local background surface can be fitted from adjacent substrate point clouds. After background separation, structural point clouds protruding from the substrate are retained as candidate SSAN point clouds.

The candidate point clouds are then denoised to remove outliers, random noise, and residual points unrelated to the attachment structures. They are clustered and segmented according to spatial distribution and distance constraints to generate multiple independent point-cloud clusters. For an SSAN composed of several adjacent components, clustering must preserve the spatial integrity of its internal components and avoid erroneous merging between adjacent SSANs.

Finally, the candidate clusters are screened and verified according to spatial connectivity, protruding geometry, and structural dimensions. The resulting SSAN point-cloud clusters provide the input for spatial indexing, component-level geometric representation and checking, and end-connection positioning-feature extraction.

#### 3.2.2. Geometric Representation of SSANs

**Figure 7:**
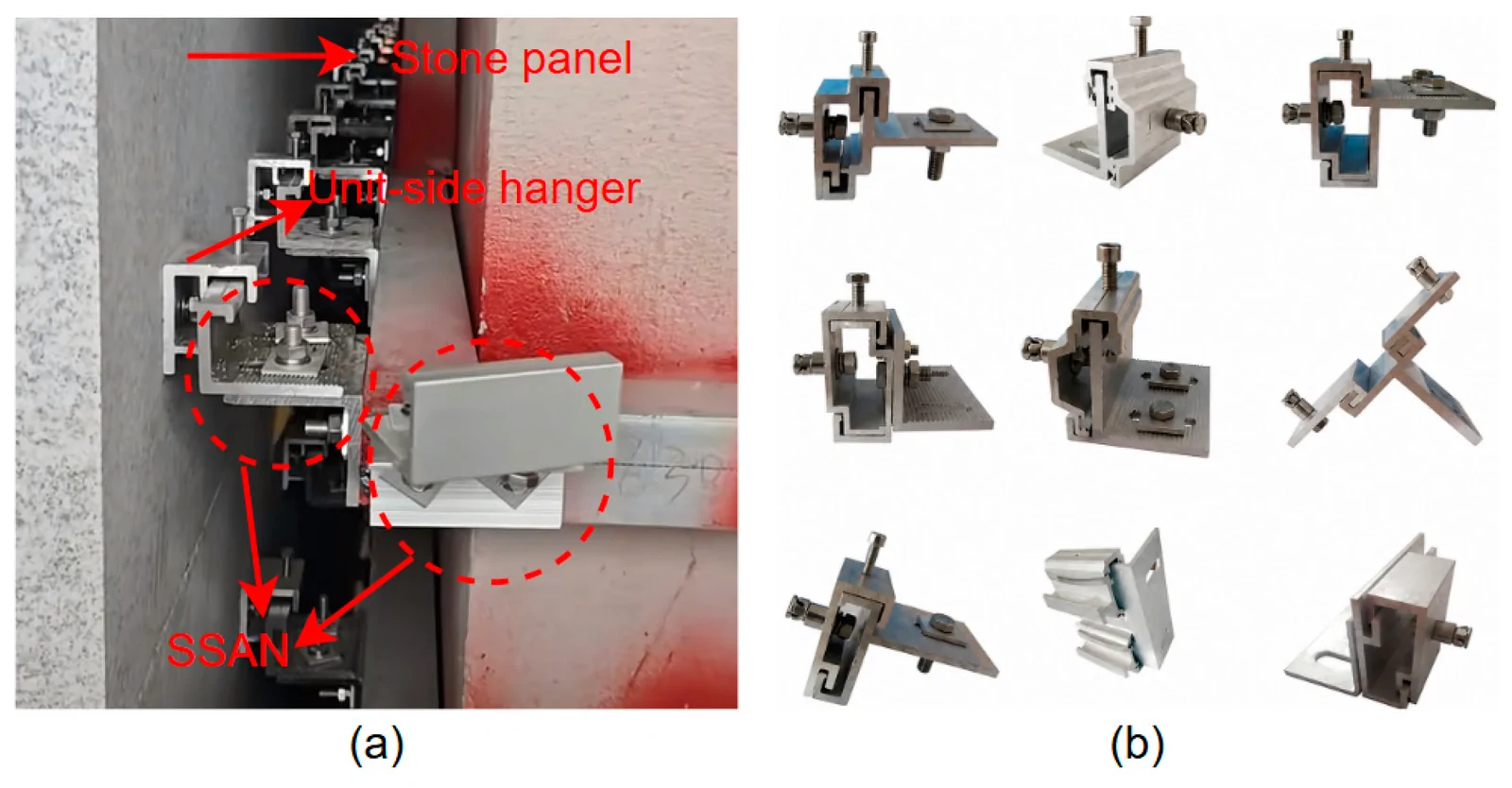
Stone curtain wall attachment system: (**a**) installed attachment interface; (**b**) assemblies of unit-side hangers and SSANs.

SSANs are discretely arranged according to curtain wall divisions and on-site installation sequences. On regular planar facades, their arrangement generally follows distinct row-and-column relationships. On curved or irregular facades, SSANs may instead be arranged along surface directions, inclined divisions, or spatial paths. The attachment interface and component assemblies of a stone curtain wall attachment system are shown in Figure 7.

**Figure 8:**
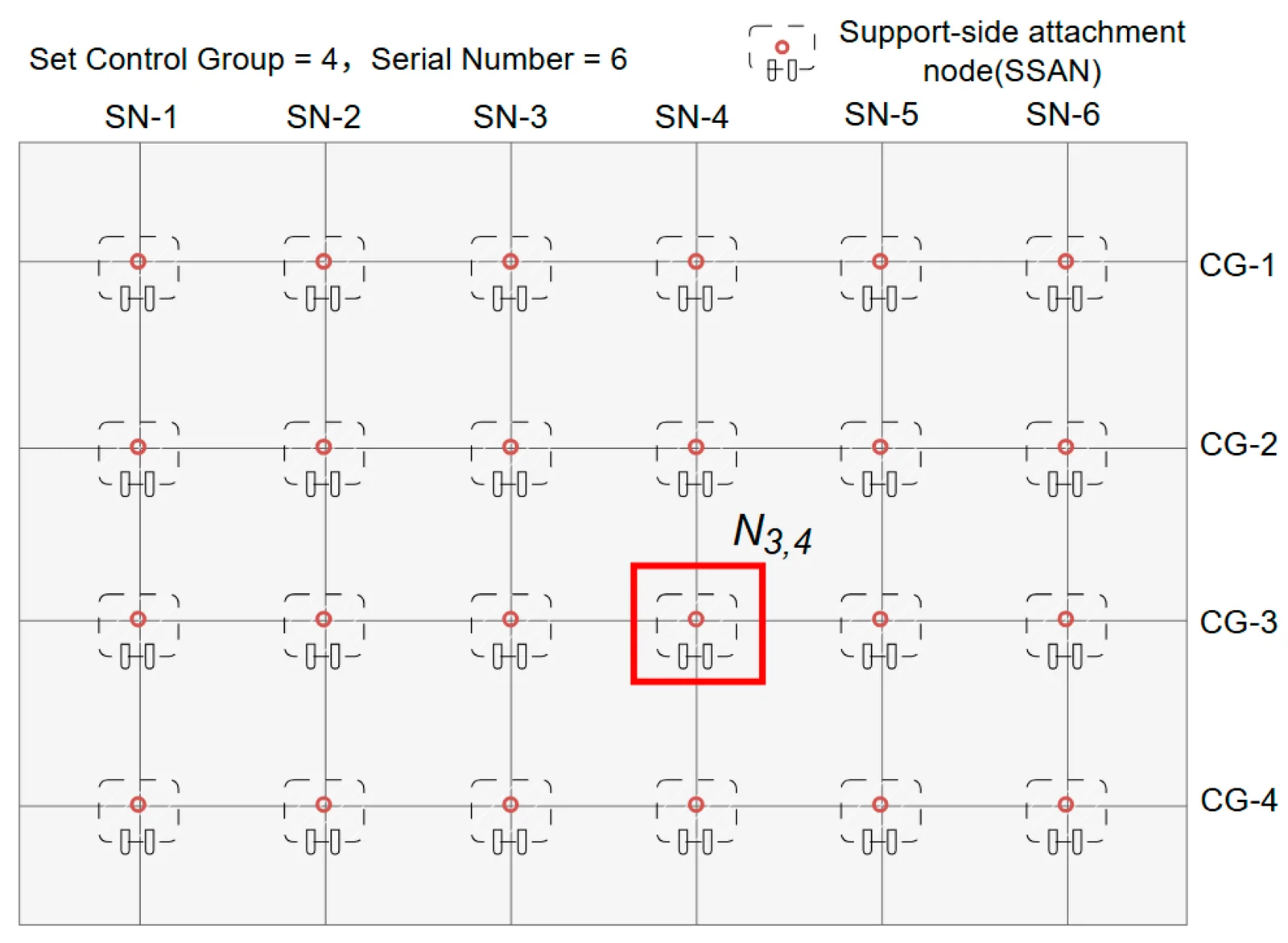
Logical indexing of SSANs.

To represent the layout relationships among SSANs, spatial logical indexing is established using control groups and node serial numbers within each group. A control group contains SSANs that follow the same arrangement direction or installation sequence. The node serial number identifies the position of an SSAN within that sequence. The SSAN set is expressed in Equation (3):

*N* = {*N_c_*_,*s*_} (3)

where $N_{c,s}$ denotes the SSAN indexed by (*c*,*s*); *c* identifies the control group, and *s* identifies the node serial number within that group. The values of *c* and *s* are determined by the actual arrangement of installed SSANs within the inspection area. The index (*c*,*s*) represents the logical position of each SSAN within the measured layout system, as shown in Figure 8.

**Figure 9:**
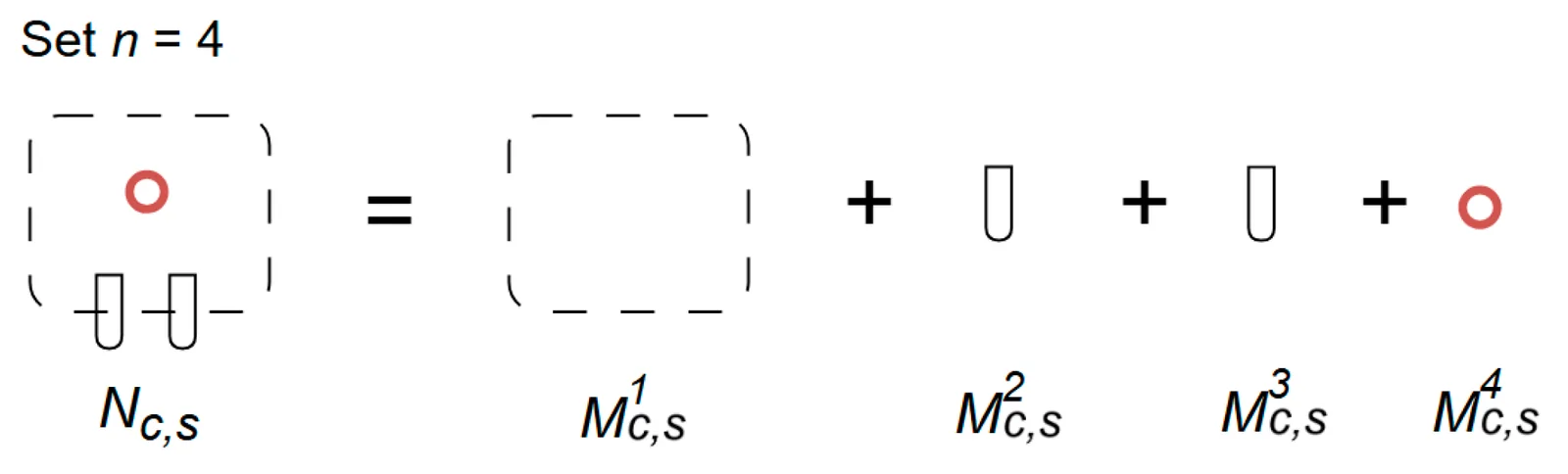
Internal component composition of an SSAN.

The number and cross-sectional geometry of internal components depend on the specific attachment configuration. After spatial indexing is established, the internal component set of an individual SSAN can be expressed in Equation (4):

(4) $$N_{c,s}=\left\{M_{c,s}^{1},M_{c,s}^{2},\ldots ,M_{c,s}^{n}\right\}$$

where $M_{c,s}^{i}$ denotes the internal component indexed by *i* within SSAN $N_{c,s}$. The indices *c* and *s* are inherited from the spatial logical index of the SSAN, and *n* is the number of internal components to be checked. The internal component representation is shown in Figure 9.

#### 3.2.3. “Point–Line–Plane”-Based Component-Level Geometric Checking

**Figure 10:**
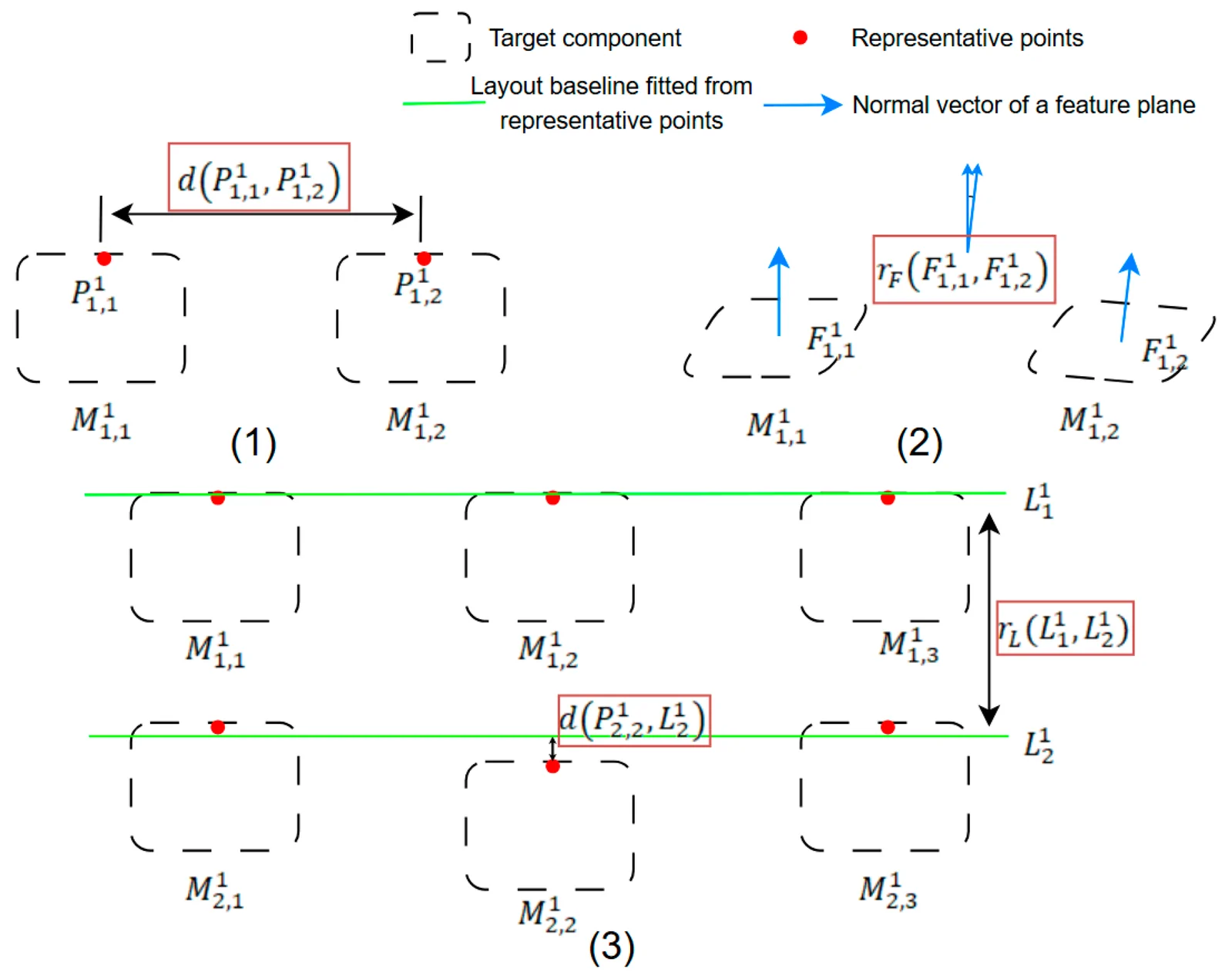
Component-level geometric relationships.

The measured data of SSANs are mainly obtained from point clouds of exposed component surfaces. Internal solid geometry and concealed connection relationships are generally difficult to observe directly. To convert different attachment configurations into computable installation checking objects, representative points, feature planes, and layout baselines are used to describe the observable geometric features of internal components of SSANs. Representative points characterize component positions, feature planes characterize local orientations or installation references, and layout baselines characterize the sequential relationships formed by same-type components within a control group. Figure 10 illustrates how these geometric primitives support point-to-point, point-to-line, line-to-line, and plane-to-plane checking.

The point and plane geometric primitives of internal component $M_{c,s}^{i}$ within SSAN $N_{c,s}$ are expressed in Equation (5):

(5) $$M_{c,s}^{i}=\left\{P_{c,s}^{i},F_{c,s}^{i}\right\}$$

where $P_{c,s}^{i}$ is the representative point of internal component $M_{c,s}^{i}$. It may be defined by the component center, cross-sectional center, geometric centroid, boundary feature point, or a predefined positioning point and is used to characterize the positional state of the component. $F_{c,s}^{i}$ is the feature plane of $M_{c,s}^{i}$. It may be determined from an observable component surface, installation reference plane, bearing surface, or locally fitted plane. Its normal vector characterizes the orientation of the component.

Representative points of same-type components within the same control group are further used to construct a layout baseline. For components of the same type indexed by *i* within control group *c*, the corresponding layout baseline is expressed in Equation (6):

(6) $$L_{c}^{i}=\mathrm{Line}\left(P_{c,1}^{i},P_{c,2}^{i},\ldots P_{c,S_{c}}^{i}\right)$$

where $L_{c}^{i}$ denotes the layout baseline associated with same-type components indexed by *i* in control group *c*, and *Line*(·) is a function that generates the baseline from their representative points. Depending on the facade geometry and SSAN arrangement, $L_{c}^{i}$ may be a straight line, polyline, spatial curve, or discrete node path.

For large-scale or geometrically complex facades, these layout relationships can be established in local surface-based coordinate systems. In such cases, polylines or spatial curves may be used to follow the facade geometry, while local tangent directions and feature-plane normals can be used to describe the relative position and orientation of neighboring components. Accordingly, the geometric checking relationships can be extended to curve-based or locally piecewise representations when required by the facade geometry.

After spatial indexing, internal component sets, and geometric primitives are established, installation checking relationships can be defined among same-type components. For the same-type components in different SSANs, the checking criteria mainly include relative component positions, deviations from the layout baseline within a control group, relative arrangements between different control groups, and differences in component orientation. These criteria are represented by point-to-point distance, point-to-line distance, line-to-line relationship, and plane-to-plane relationship, respectively.

For the same-type components with component index *i*, the set of installation checking relationships is expressed in Equation (7):

(7) $$R^{i}=\left\{d\left(P,P\right),d\left(P,L\right),r_{L}\left(L,L\right),r_{F}\left(F,F\right)\right\}$$

where $R^{i}$ denotes the set of component-level measured checking relationships corresponding to component index *i*. *d*(*P*,*P*) denotes the distance between representative points and is used to check the relative positions of same-type components. *d*(*P*,*L*) denotes the deviation of a representative point from its corresponding layout baseline and evaluates the deviation of an internal component from the measured layout reference of the SSAN group. $r_{L}\left(L,L\right)$ denotes the relative relationship between corresponding layout baselines in different control groups and may include inter-line distance, included angle, parallelism, or inter-curve deviation. $r_{F}\left(F,F\right)$ denotes the orientation relationship between corresponding feature planes and may include plane distance, angle between normal vectors, parallelism, or orientation consistency.

A subset of these relationships may be selected according to the SSAN configuration, point-cloud observability, and installation-checking requirements of a specific project. The checking results of all internal components collectively characterize the measured installation states of the SSANs and provide the basis for end-connection positioning-feature extraction and subsequent tracing of out-of-range items.

The specific geometric primitives used for checking are determined according to the observable geometry and functional role of each component. Although component shapes and connection forms may vary among different curtain wall attachment systems, the spatial indexing scheme and the use of representative points, layout baselines, and feature planes provide a common geometric representation. The corresponding feature definitions and dimensional constraints can then be selected according to the actual attachment configuration.

#### 3.2.4. End-Connection Positioning Feature Extraction

The end-connection positioning feature is a local geometric feature of the end-connection component that directly contacts the unit-side hanger and determines its seating, bearing, or limiting condition. Its form depends on the specific attachment configuration and may be represented by a limiting slot, bearing edge, positioning surface, or attachment seating center.

When an end-connection positioning feature has a complete boundary and sufficient sampling density in the point cloud, its position, boundary, or direction can be directly obtained through local point cloud segmentation, boundary extraction, and geometric fitting. For small end features, incomplete boundaries, or unstable point cloud representations, stable and visible representative points, feature planes, or boundary lines of the end-connection component are first extracted. The spatial position of the positioning feature is then inferred from its fixed structural relationship with the component reference edge or reference plane.

The extracted end-connection positioning features are used to establish distance, direction, and orientation relationships within the corresponding SSAN group. They also provide the input for subsequent multi-point attachment geometric compatibility assessment and unit-side hanger pre-adjustment calculation.

### 3.3. Curtain Wall Unit Installation Compatibility Assessment and Hanger Adjustment

Before lifting, the stone panels, sub-frame, and unit-side hangers of each curtain wall unit have already been preassembled. According to the curtain wall design, each unit has a relatively fixed hanger array. Its installation compatibility therefore depends on the multi-point geometric relationship between the unit-side hanger array and the support-side end-connection positioning features. Based on the SSAN end-connection positioning features extracted in Section 3.2.4, this section establishes the mapping between a curtain wall unit and its corresponding SSAN group, evaluates multi-point attachment geometric compatibility, checks the overall pose and joint-width coordination of the curtain wall unit, and outputs hanger pre-adjustment parameters and installation decisions.

#### 3.3.1. Mapping Between Curtain Wall Unit Hangers and SSANs

To convert support-side end-connection positioning features into unit-side hanger pre-adjustment parameters, correspondences must be established among the curtain wall unit, the unit-side hanger array, and the SSAN group. Let *U_p_*_,*q*_ denote the curtain wall unit indexed by (*p*,*q*), where *p* identifies the curtain wall unit arrangement strip and *q* identifies the unit serial number within that strip, and let *Ω_p_*_,*q*_ denote the corresponding SSAN. The composition of *Ω_p_*_,*q*_ is determined by the actual arrangement of SSANs and their attachment relationships with the curtain wall unit. It represents the correspondence among the curtain wall unit, the local positions of unit-side hangers, and the logical indices of SSANs.

For a curtain wall unit with an *α* × *β* local hanger layout in a regularly and continuously arranged region, *Ω_p_*_,*q*_ is expressed using the SSAN index *N_c_*_,*s*_ as shown in Equation (8):

(8) $$\Omega_{p,q}={\left[N_{\left(p-1\right)\alpha +a,\;\left(q-1\right)\beta +b}\right]}_{\alpha \times \beta },a=1,\ldots ,\alpha ,b=1,\ldots ,\beta$$

where *Ω_p_*_,*q*_ is the ordered SSAN group corresponding to curtain wall unit *U_p_*_,*q*_, and *a* and *b* denote the local indices within the group corresponding to unit-side hangers. For the SSAN at local position (*a*,*b*) in *Ω_p_*_,*q*_, its global index is determined by:

*c* = (*p* − 1)*α* + *a*, *s* = (*q* − 1)*β* + *b*

The unit-side hanger array of curtain wall unit *U_p_*_,*q*_ is denoted by *H_p_*_,*q*_ and expressed in Equation (9):

(9) $$H_{p,q}={\left[H_{a,b}\right]}_{\alpha \times \beta },\;a=1,\;\ldots ,\alpha ,\;b=1,\ldots ,\beta$$

Its array form is:

$$H_{p,q}=[\begin{array}{cccc} H_{1,1} & H_{1,2} & \ldots & H_{1,\beta }\\ H_{2,1} & H_{2,2} & \ldots & H_{2,\beta }\\ \vdots & \vdots & \ddots & \vdots \\ H_{\alpha ,1} & H_{\alpha ,2} & \ldots & H_{\alpha ,\beta } \end{array}]$$

where *H_a_*_,*b*_ denotes the unit-side hanger at local index (*a*,*b*) within curtain wall unit *U_p_*_,*q*_. Because *Ω_p_*_,*q*_ and *H_p_*_,*q*_ use the same local indexing scheme, a SSAN and a unit-side hanger with the same local index (*a*,*b*) form a corresponding attachment pair.

**Figure 11:**
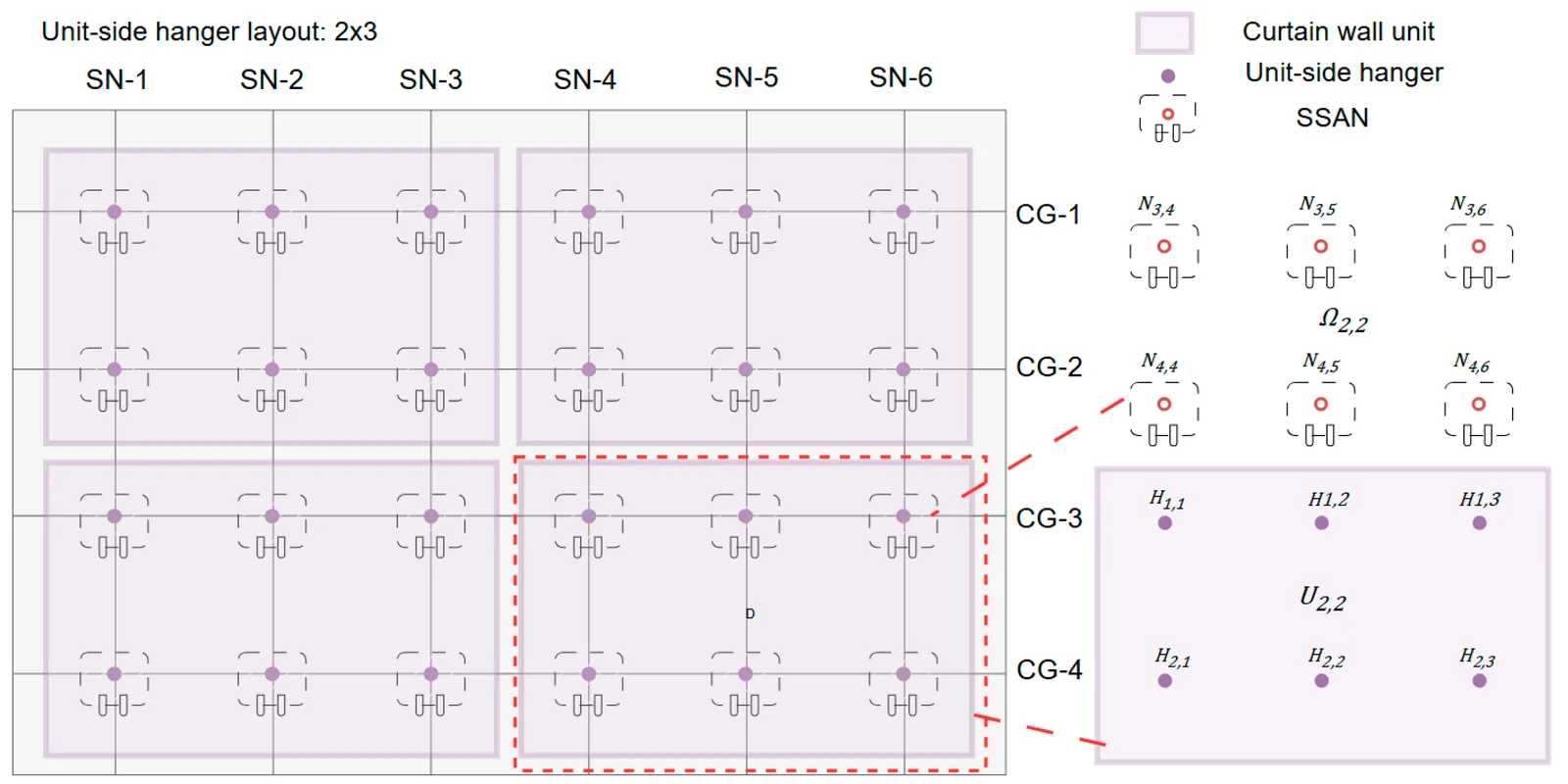
Mapping among curtain wall units, unit-side hangers, and SSANs. The red dashed box indicates a single curtain wall unit selected for illustrating the corresponding hanger–SSAN mapping.

Using a curtain wall unit with *α* = 2 and *β* = 3 as an example, Figure 11 illustrates the mapping among *U*_2,2_, *Ω*_2,2_, and the unit-side hanger array.

#### 3.3.2. Multi-Point Attachment Geometric Compatibility Assessment

After the mapping among curtain wall unit *U_p_*_,*q*_, SSAN group *Ω_p_*_,*q*_, and unit-side hanger array *H_p_*_,*q*_ is established, the measured geometric relationships among the end-connection positioning features in *Ω_p_*_,*q*_ can be converted into target layout relationships of the unit-side hangers.

Multi-point attachment compatibility requires not only that each hanger engage with its corresponding SSAN, but also that the relative distances, directions, and local orientations within the hanger array conform to the measured states of the support-side end-connection positioning features.

For any two SSANs *N_c_*_,*s*_ and *N_c′_*_,*s′*_ in *Ω_p_*_,*q*_ that have an installation control relationship, their end-connection positioning features are represented by $P_{c,s}^{n}$ and $P_{\mathrm{c'},\mathrm{s'}}^{n}$, respectively. Their corresponding unit-side hangers are *H_a_*_,*b*_ and *H_a′_*_,*b′*_. The target layout distance between the unit-side hangers is determined by the measured distance between the support-side end-connection positioning feature points, as shown in Equation (10):

(10) $$d\left(H_{a,b},H_{a^{\prime },b^{\prime }}\right)=d\left(P_{c,s}^{n},P_{c^{\prime },s^{\prime }}^{n}\right)$$

where $d\left(P_{c,s}^{n},P_{c^{\prime },s^{\prime }}^{n}\right)$ is the measured distance between the support-side end-connection positioning feature points, and $d\left(H_{a,b},H_{a^{\prime },b^{\prime }}\right)$ is the target layout distance between the corresponding unit-side hangers.

**Figure 12:**
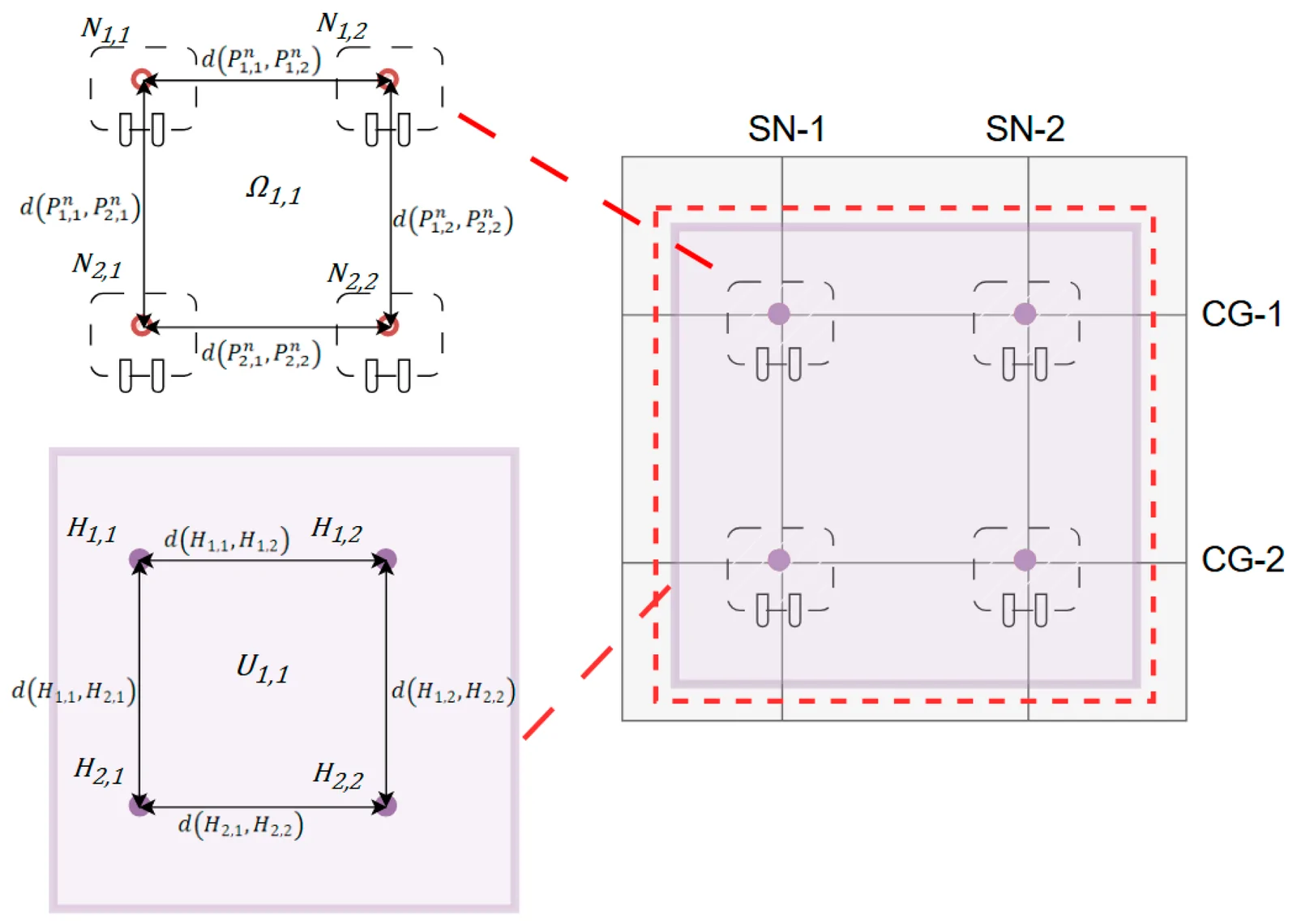
Multi-point attachment compatibility assessment. The red dashed box indicates the selected curtain wall unit, and the red dashed lines link it to the corresponding SSAN group and unit-side hanger layout shown on the left.

Depending on the hanger arrangement and installation control requirements, the controlled relationships may include relationships between adjacent hangers within the same local control group, relationships between hangers with the same serial number in different local control groups, and key hanger relationships that affect the overall unit boundary. Figure 12 illustrates the mapping from measured relationships among support-side end-connection positioning features to target layout relationships among unit-side hangers.

After target hanger layout relationships are obtained, their feasibility must be evaluated against the effective adjustment range of the hanger configuration. This range is jointly determined by the dimensions of the unit-side hangers, slot lengths, connection-hole positions, sub-frame boundaries, and local component arrangement.

For a controlled geometric relationship *r*, the effective allowable interval can be expressed as

(11) $$\Omega_{r}^{\left(q\right)}=\Omega_{H,r}\cap \Omega_{S,r}^{\left(q\right)}\cap \Omega_{B,r}$$

where *q* denotes the SSAN configuration, Ω*_H_*_,*r*_ is the range achievable through unit-side hanger adjustment, $\Omega_{S,r}^{\left(q\right)}$ is the admissible range imposed by the geometry and connection characteristics of the corresponding SSAN configuration, and Ω*_B_*_,*r*_ represents the design-boundary or joint-width constraint. A measured target relationship is considered adjustable when it falls within $\Omega_{r}^{\left(q\right)}$. For a different SSAN configuration, the hanger-side adjustment model can be retained, while $\Omega_{S,r}^{\left(q\right)}$ is redefined according to the corresponding end-connection geometry and attachment mechanism.

If a target layout relationship falls within the effective adjustment range, it is included in the hanger pre-adjustment output. If it exceeds the effective adjustment range, the corresponding hanger relationship is marked as an out-of-range item, and the associated SSAN indices *N_c_*_,*s*_ and *N_c′_*_,*s′*_ are recorded. The associated SSANs are flagged for field rechecking to determine whether support-side adjustment is required.

In the subsequent installation decision output, these indices are linked to the component-level measured checking results $R^{i}$. The corresponding (*c*,*s*) indices can therefore be used to trace the positional, layout-baseline, or orientation deviations of internal components within the corresponding SSANs.

#### 3.3.3. Overall Pose and Joint-Width Coordination of Curtain Wall Units

After multi-point attachment geometric compatibility is confirmed, the hanger pre-adjustment results must be coordinated within the overall boundary system of the curtain wall unit. Unit-side hangers can be locally pre-adjusted according to the support-side end-connection positioning features. However, an overall offset of the adjusted hanger array relative to the curtain wall unit control edges may cause nonuniform joint widths, surface misalignment, or occupation of the installation space required by adjacent units. Hanger pre-adjustment calculation must therefore also account for the overall pose of the curtain wall unit and joint-width coordination.

The boundary positioning distance *d_b_*(*U_p_*_,*q*_,*a*) represents the distance between the hanger reference position associated with local control group *a* in curtain wall unit *U_p_*_,*q*_ and the corresponding unit design control edge. This distance is determined from the measured coordinates of the support-side end-connection positioning features, the corresponding local design control edge, and the structural relationship of the unit-side hangers.

In curved, inclined, or irregularly divided regions, the design control edges corresponding to different local control groups may not be at the same elevation. Therefore, *d_b_*(*U_p_*_,*q*_,*a*) is calculated separately for the local design control edge corresponding to each local control group.

**Figure 13:**
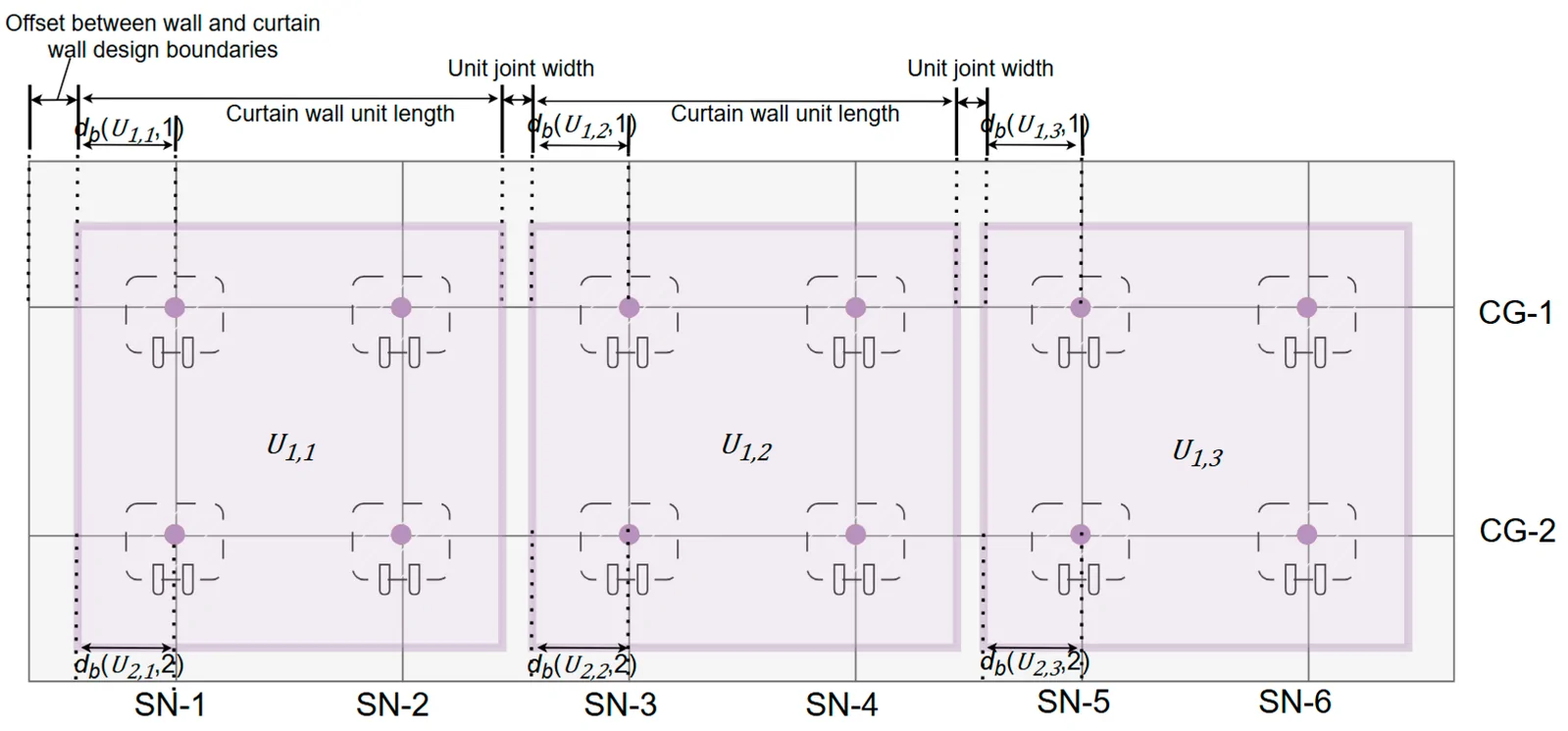
Geometric relationships among curtain wall unit boundaries, hanger positions, and joint widths. The dashed symbols represent SSANs, and the purple dots indicate the corresponding unit-side hanger positions.

As shown in Figure 13, continuously installed curtain wall units also involve relationships among unit length, joint width, and adjacent installation boundaries. The boundary positioning distance of the current unit can be determined directly from the corresponding end-connection positioning feature and design control edge. The boundary positioning distance of an adjacent unit can be calculated from the measured distance between the end-connection positioning features of adjacent units, the curtain wall unit dimensions, and the design joint width.

Accordingly, *d_b_*(*U_p_*_,*q*_,*a*), together with the joint-width relationship between adjacent units, forms the input for overall pose coordination of the curtain wall units.

#### 3.3.4. Hanger Pre-Adjustment and Installation Decision Output

The hanger pre-adjustment and installation decision output converts the measured checking results of SSANs into implementable unit-side hanger layout parameters and support-side rechecking indices before lifting. Curtain wall unit *U_p_*_,*q*_ is used as the basic output object. The measured relationships among support-side end-connection positioning features, the adjustment range of unit-side hangers, the boundary positioning distance *d_b_*(*U_p_*_,*q*_,*a*), and the joint-width coordination results are jointly considered to generate pre-adjustment parameters and rechecking decisions for on-site installation.

**Figure 14:**
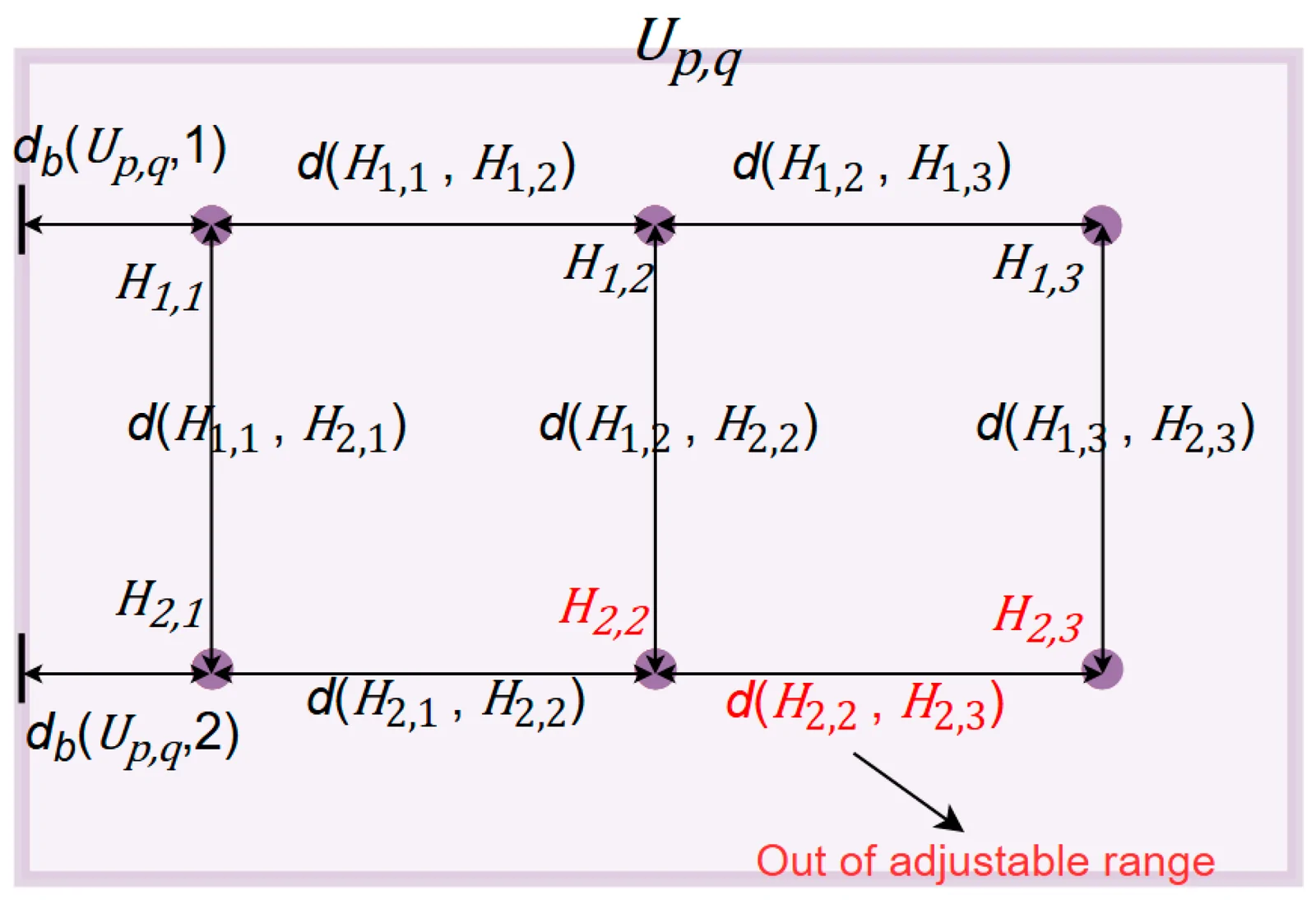
Hanger layout parameters and an example of an out-of-range relationship.

As shown in Figure 14, the output parameters mainly include two types of relationships. The first consists of target layout relationships among unit-side hangers and constrains the relative geometry within the hanger array. The second is the boundary positioning distance *d_b_*(*U_p_*_,*q*_,*a*), which controls the positional relationship between the hanger array and the design control edge of the curtain wall unit.

When all calculated relationships fall within the hanger adjustment range and design tolerance, the installation decision is to lift the curtain wall unit after hanger pre-adjustment. When an out-of-range relationship exists, a support-side rechecking and adjustment decision is generated, together with the associated out-of-range SSANs.

When an out-of-range relationship is identified, the corresponding SSAN index *N_c_*_,*s*_ is recorded. If the relationship involves two SSANs, both *N_c_*_,*s*_ and *N_c′_*_,*s′*_ are recorded. These indices are linked to the corresponding component-level geometric checking results $R^{i}$ established in Section 3.2.3, thereby enabling the positional, layout-baseline, or orientation deviations of internal components of the SSANs to be traced.

Through this process, the measured geometric states of SSANs are converted into unit-side hanger pre-adjustment parameters and installation decisions, forming a closed workflow from SSAN checking and hanger pre-adjustment to on-site installation execution.

## 4. Case Study

### 4.1. Project Overview

**Figure 15:**
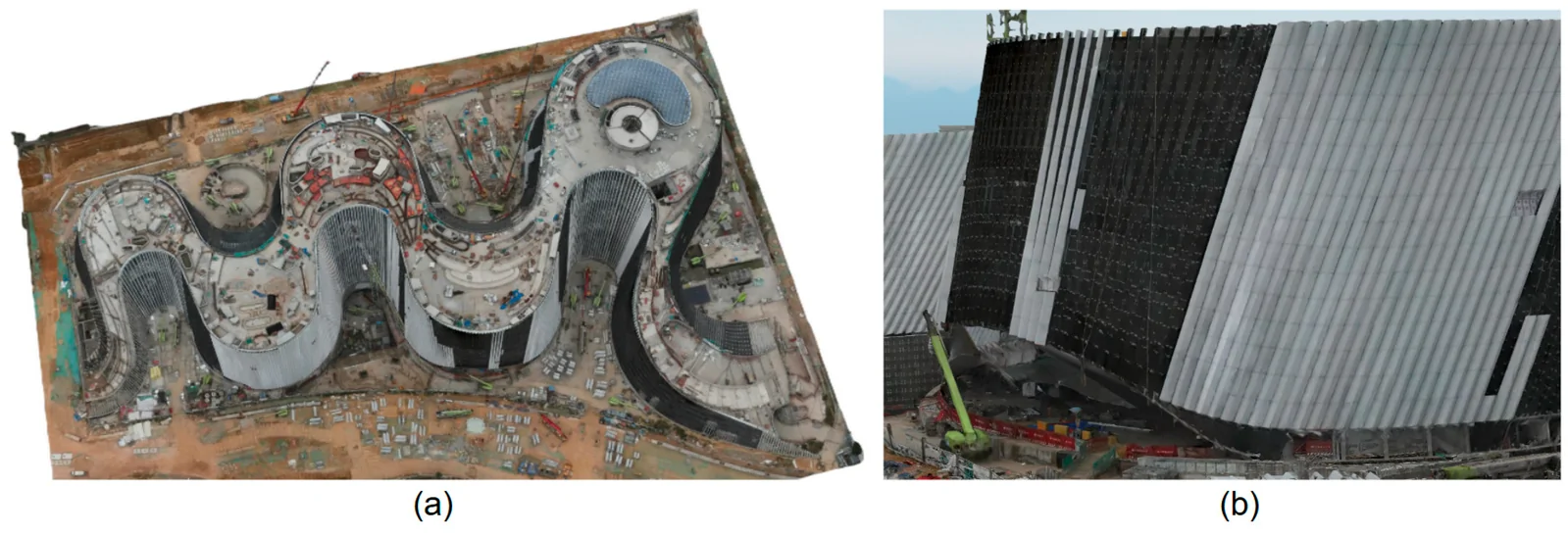
3D models of the case project: (**a**) experimental site; (**b**) curtain wall installation area.

To validate the proposed method for SSAN checking and hanger pre-adjustment in multi-point attachment unit systems of dry-hanging stone curtain walls, a stone curtain wall installation project in a large public cultural building under construction in Shenzhen was selected as a case study. The building has a large-scale steel structural system, and parts of its facade adopt a multi-point attachment unit system for dry-hanging stone curtain walls. The SSANs had been installed before the lifting of the curtain wall units. Therefore, installation checking and hanger pre-adjustment were required before lifting according to the measured states of the SSANs. The UAV oblique photogrammetric model of the experimental site and the local 3D model of the curtain wall installation area are shown in Figure 15.

**Figure 16:**
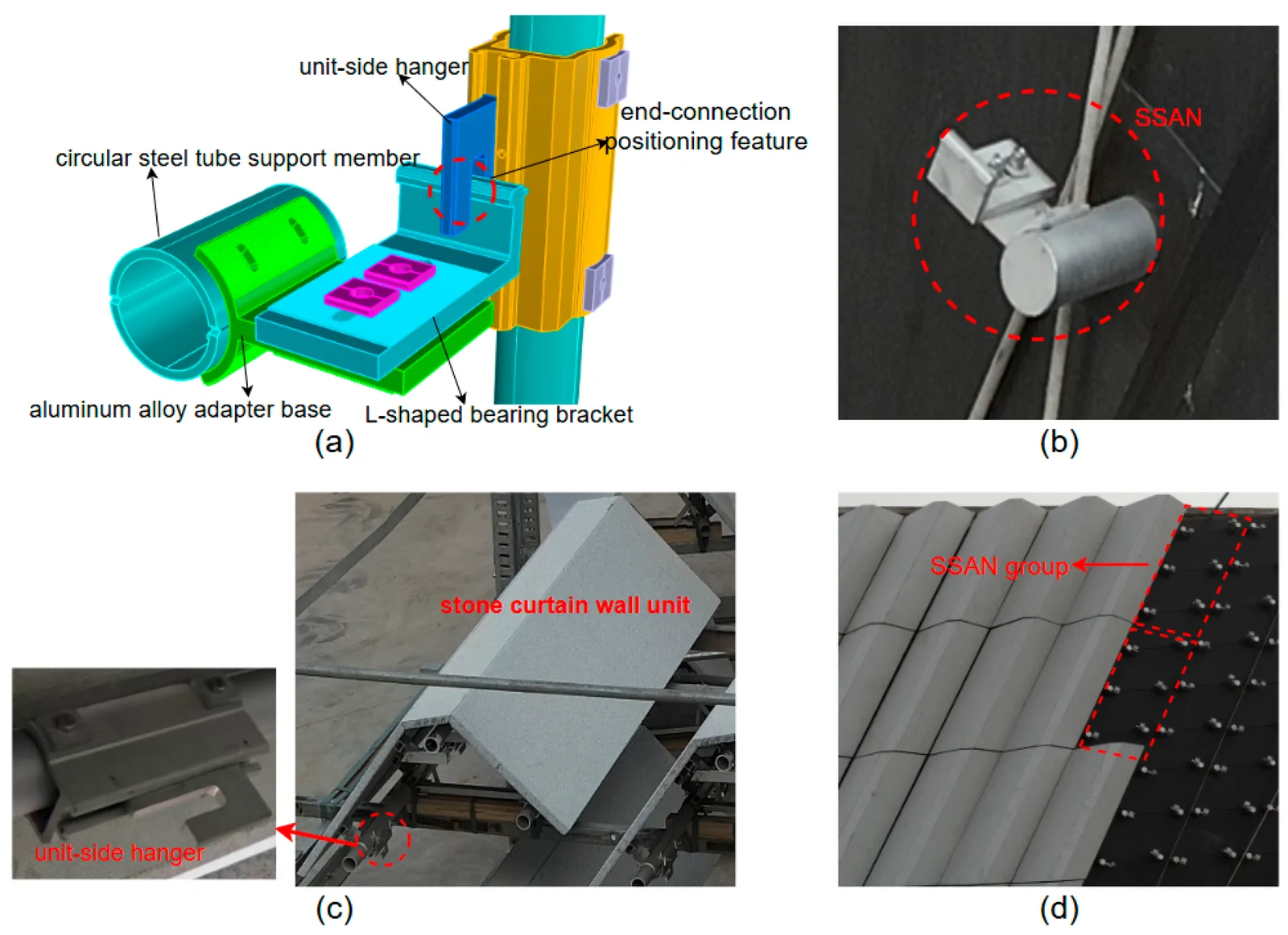
Multi-point attachment structure of the case curtain wall unit: (**a**) attachment structure; (**b**) SSAN; (**c**) stone curtain wall unit and hanger; (**d**) multi-point attachment installation.

In this project, each SSAN mainly consists of a circular steel tube support member, an aluminum alloy adapter base, and an L-shaped bearing bracket. Each curtain wall unit consists of stone panels, a rear aluminum alloy frame, and unit-side aluminum alloy hangers. Six unit-side hangers are arranged in two local control groups, with three serially numbered hangers in each group, and correspond to six SSANs. During installation, the unit-side aluminum alloy hangers form multi-point attachment relationships with the end-connection positioning features of the L-shaped bearing brackets. The SSAN and curtain wall unit configurations are shown in Figure 16.

### 4.2. Data Acquisition and Point Cloud Fusion

#### 4.2.1. Data Acquisition and Model Reconstruction

**Figure 17:**
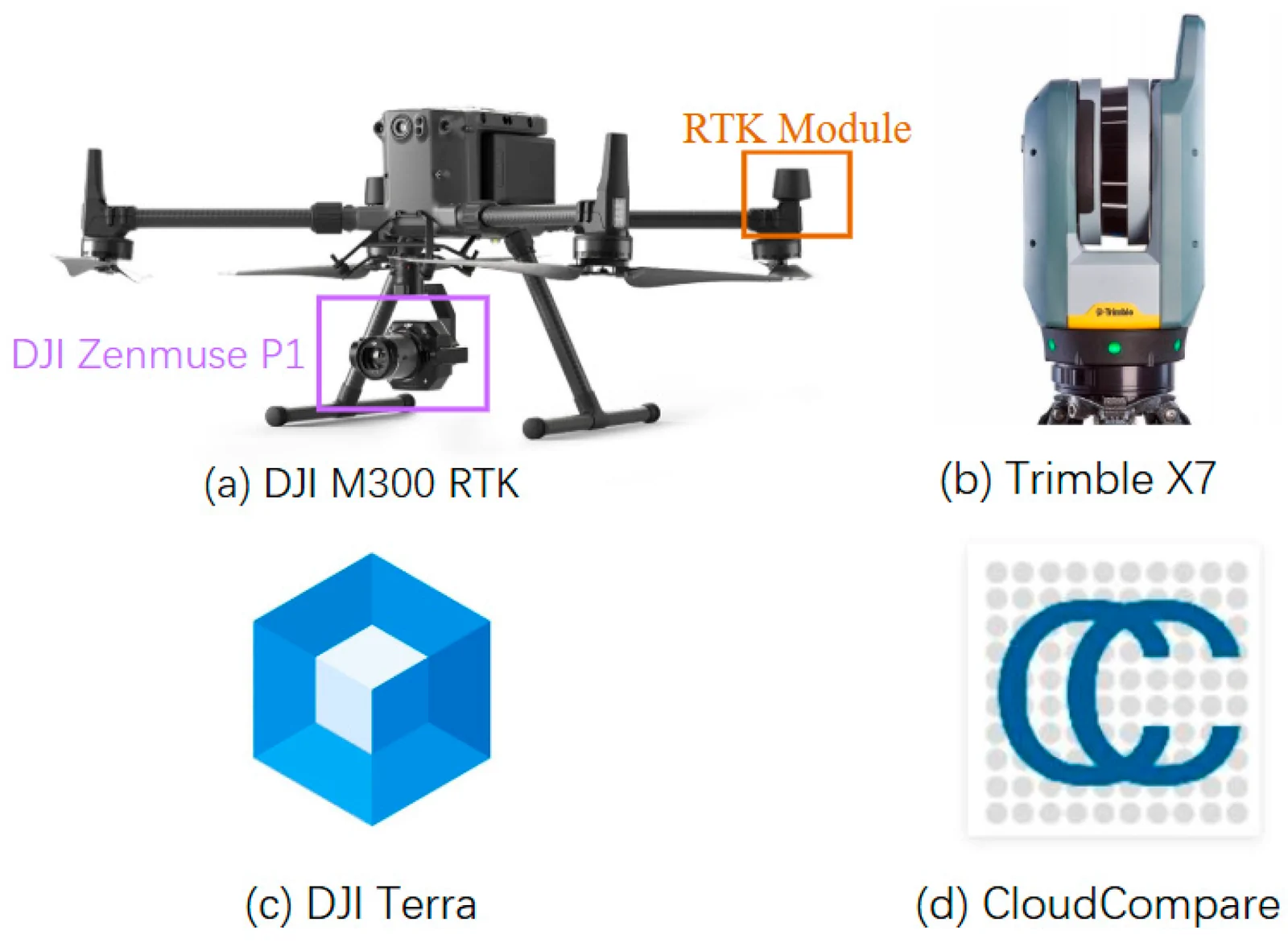
Equipment and software used in the experiment.

The hardware and software platforms used for data acquisition and processing are shown in Figure 17, and their specifications are listed in Table 1.

A DJI M300 RTK UAV was used to collect oblique images of the construction site, and DJI Terra was used to reconstruct the three-dimensional oblique photogrammetric environmental model. Based on this model, the spatial location of the curtain wall inspection area, accessible positions for ground-based scanning, and safety boundaries for UAV close-range photogrammetry were identified. TLS stations and UAV close-range photogrammetry viewpoints were then planned.

**Figure 18:**
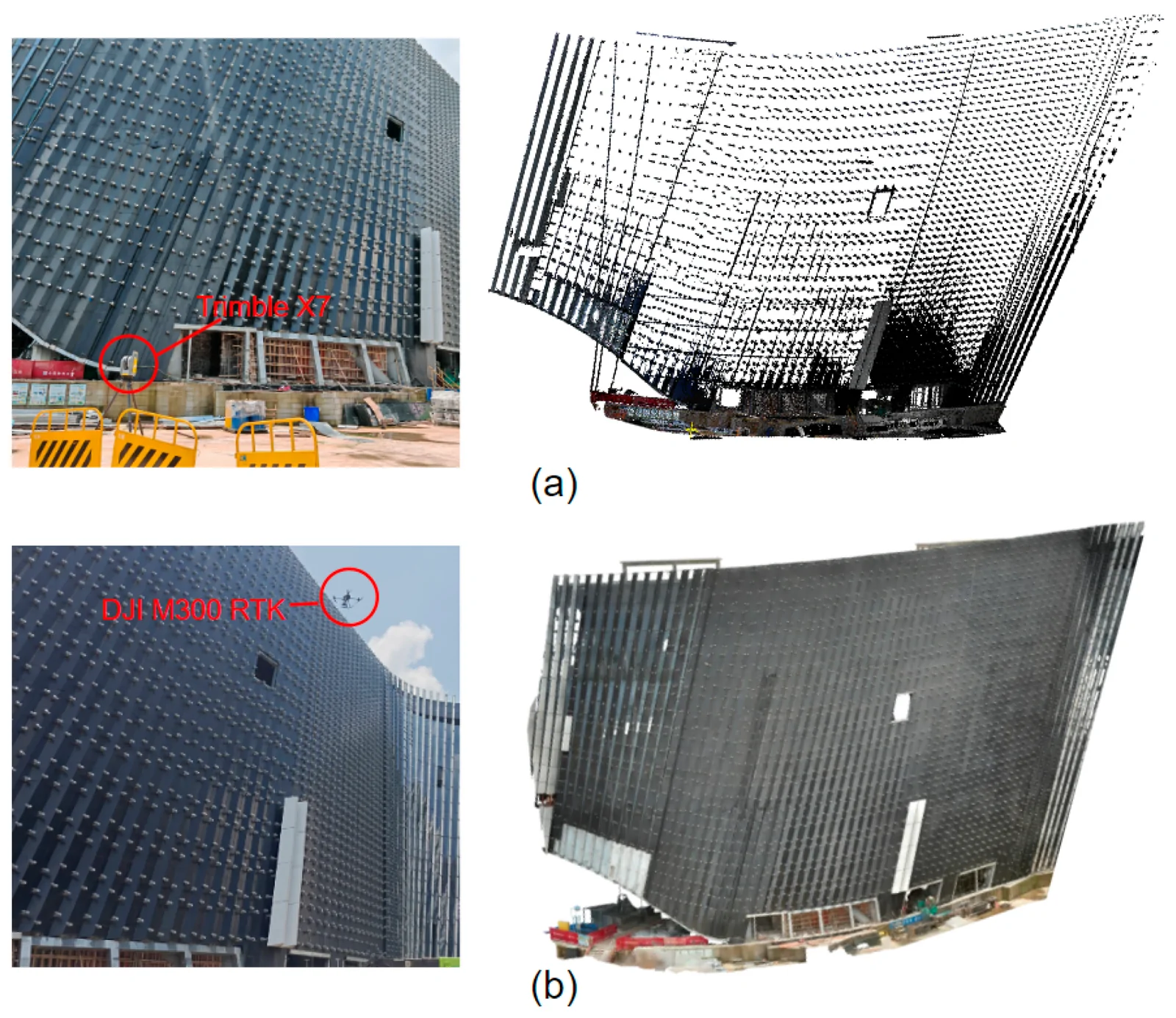
Data acquisition and 3D models of the curtain wall inspection area: (**a**) TLS and point cloud; (**b**) UAV close-range photogrammetry and 3D model.

For TLS data acquisition, stations were arranged according to the field of view and effective scanning distance of the Trimble X7, together with on-site occlusion conditions. A TLS point cloud covering the curtain wall inspection area was subsequently acquired, as shown in Figure 18a. For UAV close-range photogrammetry, the target GSD was set to 1 mm based on the DJI Zenmuse P1 camera parameters. According to Equations (1) and (2), the perpendicular shooting distance from the camera center to the target surface was calculated as *D* = 7.95 m. The horizontal and vertical coverage of a single image on the target surface were *W* = 8.15 m and *H* = 5.38 m, respectively. To provide sufficient image overlap for robust image matching and local geometric reconstruction, forward and side overlaps of 80% and 70%, respectively, were adopted. Based on these overlap settings, together with the shooting distance and camera field of view, 58 close-range viewpoints were generated, with one image acquired at each viewpoint, yielding 58 images in total. Within representative SSAN surface regions, the median surface point densities of the UAV photogrammetric and TLS point clouds were approximately 41,794 points/m^2^ and 54,151 points/m^2^, respectively. After image matching, dense point cloud reconstruction, and texture mapping, a close-range photogrammetric model of the curtain wall inspection area was obtained, as shown in Figure 18b.

#### 4.2.2. Registration Accuracy and Fusion Results

**Figure 19:**
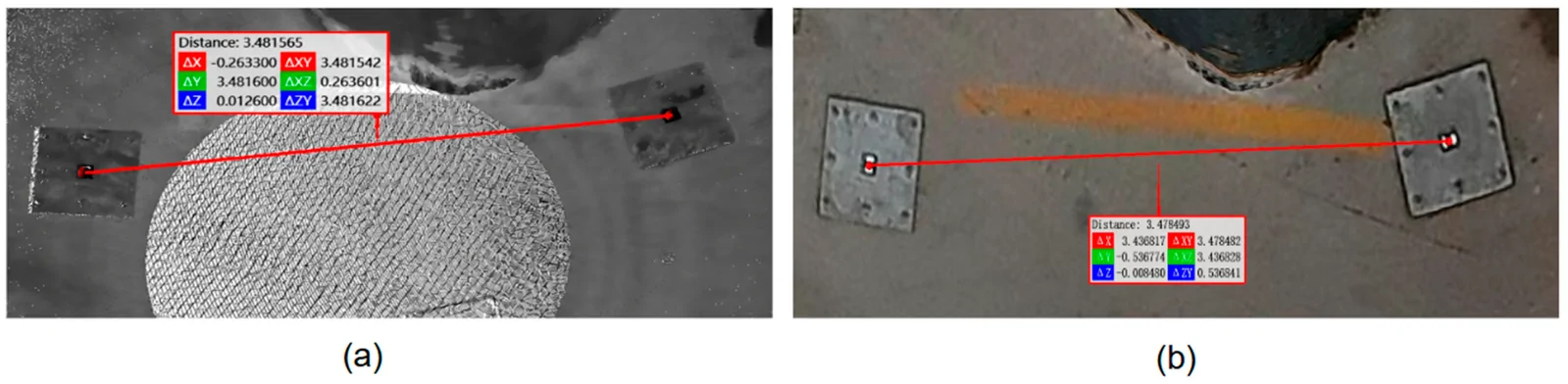
Local scale consistency between TLS and UAV data: (**a**) TLS point cloud; (**b**) UAV close-range photogrammetric model point cloud.

The geometric consistency of the UAV close-range photogrammetric model was evaluated using check targets distributed at different elevations within the curtain wall inspection area. Two targets were arranged at each evaluated floor level, and their center-to-center distances were measured separately in the TLS and UAV datasets. The targets were excluded from transformation estimation and were used as check targets to evaluate geometric consistency across the five floor levels. The comparison results are summarized in Table 2. One representative target pair is shown in Figure 19, for which the corresponding spacing was approximately 3.48 m in both datasets.

As shown in Table 2, the target-spacing differences between the TLS and UAV datasets ranged from 1 to 4 mm across the five evaluated floor levels. The mean absolute discrepancies in the X, Y, and Z directions remained within 2.0, 2.3, and 1.9 mm, respectively. Across the five evaluated floor levels, neither the target-spacing differences nor the axis-wise discrepancies showed a monotonic increase with elevation.

The inspection area contained 138 SSAN components. Before registration, both datasets were manually cropped to retain the common inspection region. The TLS point cloud was used as the reference dataset, and the UAV close-range photogrammetry point cloud was registered to the TLS coordinate system using the PCL-based procedure described in Section 3.1.5. ISS keypoints and FPFH descriptors were extracted from the component-related geometric regions, and 11,920 candidate correspondences were established based on descriptor similarity and spatial constraints. RANSAC-based correspondence rejection with a threshold of 15 mm retained 72 inliers for estimating the initial transformation. The transformation was subsequently refined using multi-scale GICP with maximum correspondence distances of 50, 25, 12, and 7 mm, followed by point-to-plane ICP with a maximum correspondence distance of 6 mm.

To quantitatively evaluate the final registration consistency, residuals were calculated over the overlapping component geometries of the 138 SSAN components. For the *i*-th valid point pair, the point-to-plane residual was calculated as:

(12) $$r_{i}=|n_{i}^{T}\left(p_{i}^{\mathrm{TLS}}-Tp_{i}^{\mathrm{UAV}}\right)|$$

where $p_{i}^{\mathrm{TLS}}$ and $p_{i}^{\mathrm{UAV}}$ denote the corresponding points in the TLS and UAV datasets, respectively; *T* is the final rigid transformation matrix; and *n_i_* denotes the local surface normal. The point-to-plane root-mean-square error was calculated as:

(13) $${\mathrm{RMSE}}_{p2\mathrm{pl}}=\sqrt{\frac{1}{N}\sum_{i=1}^{N}r_{i}^{2}}$$

where *N* is the number of valid point pairs used for residual evaluation.

A total of 219,323 valid point pairs from the overlapping component geometries were included in the final evaluation. As summarized in Table 3, the point-to-point RMSE was 5.564 mm. The point-to-plane RMSE was 3.100 mm, while the median and 95th-percentile point-to-plane residuals were 2.369 mm and 6.081 mm, respectively. Because the TLS and UAV point clouds have different sampling locations and point-density distributions on the same physical surfaces, the point-to-plane residual was adopted as the primary surface-level registration indicator. Compared with direct point-to-point distances, the point-to-plane metric reduces the influence of tangential sampling differences and more directly reflects geometric disagreement normal to the component surfaces. These results demonstrate millimeter-scale registration consistency over the evaluated component regions.

**Figure 20:**
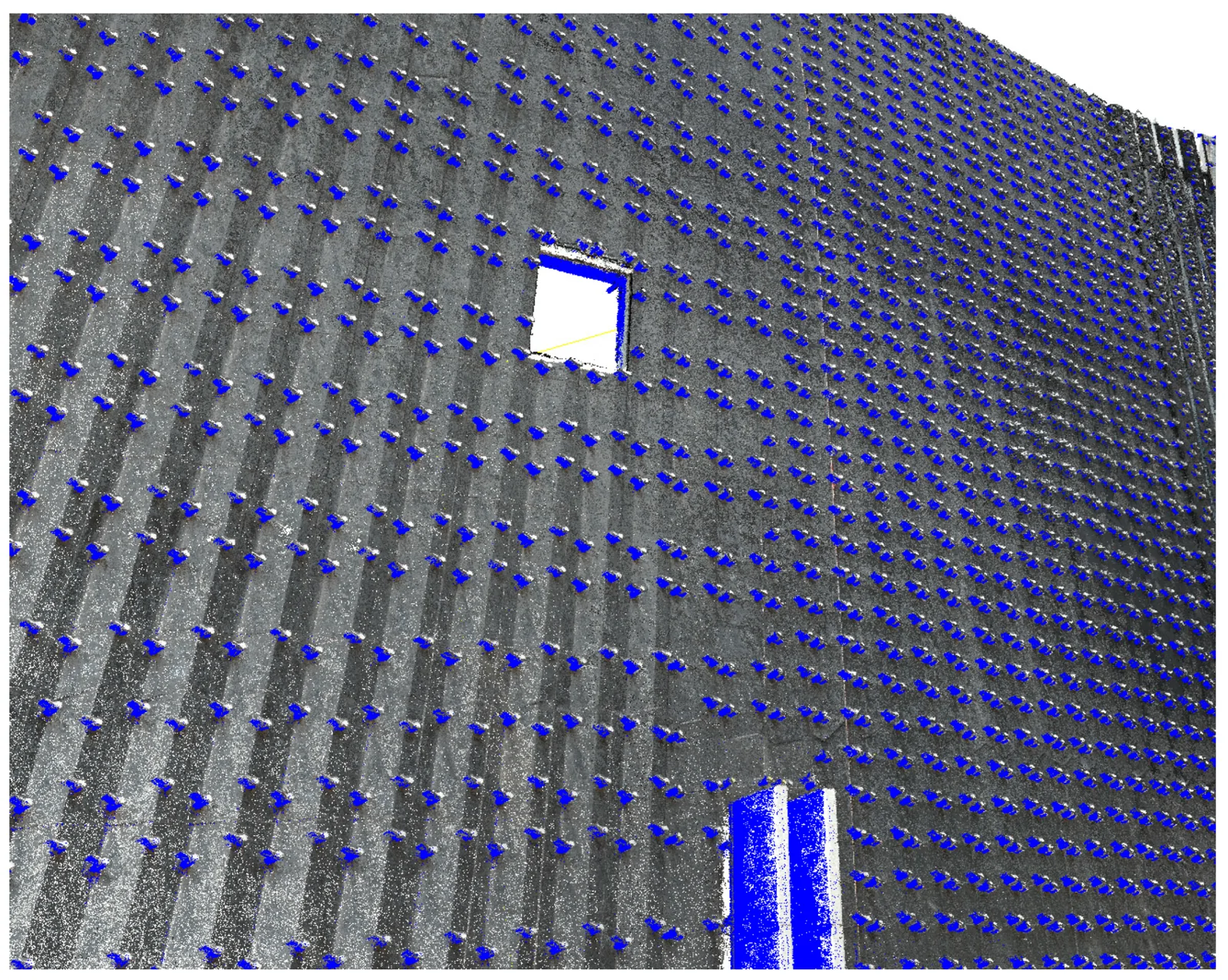
Fusion result of multi-source point clouds.

After registration, the UAV close-range photogrammetry point cloud was transformed into the TLS coordinate system and fused with the TLS dataset. The two datasets showed consistent spatial alignment at component boundaries, adapter-component outlines, and local attachment structures in the overlapping regions. The UAV data supplemented geometric information from elevated, side, and locally occluded surfaces that were insufficiently observed by terrestrial scanning, thereby improving the geometric completeness of the fused point-cloud representation. The final fusion result is shown in Figure 20.

In subsequent SSAN geometric checking and hanger pre-adjustment calculations, the TLS point cloud was retained as the primary metric reference, whereas the UAV point cloud mainly supplemented geometric information in regions with insufficient TLS visibility.

To quantitatively evaluate the additional geometric coverage provided by UAV close-range photogrammetry, a voxel-based relative geometric coverage analysis was performed within the same SSAN inspection regions. The TLS-only, UAV-only, and fused TLS–UAV point clouds were voxelized using the same voxel size. Let *V_TLS_* and *V_UAV_* denote the occupied-voxel sets of the TLS-only and UAV-only datasets, respectively, and let *V_F_* = *V_TLS_* ∪ *V_UAV_* denote the occupied-voxel set of the fused dataset. The relative geometric coverage of each individual dataset was calculated with respect to the fused observed reference as

(14) $$C_{D}=\frac{|V_{D}\cap V_{F}|}{|V_{F}|}\times 100\%,\;D\in \left\{\mathrm{TLS},\mathrm{UAV}\right\}$$

The fused dataset represents the union of the observed geometry and therefore has a relative coverage of 100%. Accordingly, this metric expresses the proportion of the fused observed geometry represented by each individual dataset.

As summarized in Table 4, the TLS-only and UAV-only datasets achieved relative geometric coverages of 68.3% and 95.2%, respectively, whereas the fused TLS–UAV dataset provided the full observed reference coverage. The results indicate that UAV photogrammetry provides broader geometric coverage than TLS alone and further complements terrestrial scanning in regions with limited visibility. The supplementary observations were mainly located on upper surfaces, lateral surfaces, and locally occluded regions, which are particularly relevant to the subsequent extraction of adapter-base and end-connection features.

**Figure 21:**
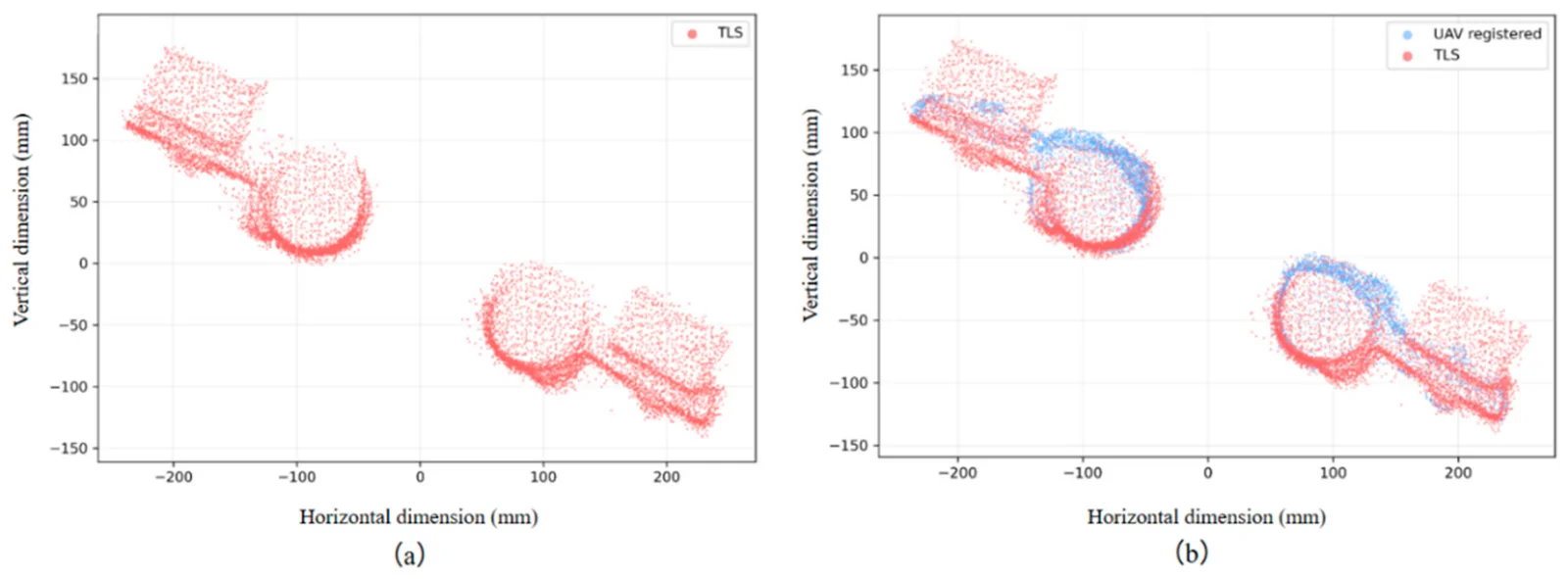
Representative comparison of SSAN point-cloud coverage: (**a**) TLS-only point cloud; (**b**) registered TLS–UAV point clouds after UAV data supplementation.

Figure 21 shows a representative SSAN for comparison. Figure 21a presents the TLS-only point cloud, while Figure 21b shows the registered TLS–UAV point clouds after UAV data supplementation. The comparison visually demonstrates the additional geometric observations provided by the UAV data and the improved completeness of the SSAN representation after fusion.

### 4.3. SSAN Extraction and Installation-State Checking

#### 4.3.1. SSAN Extraction and Index Mapping

SSANs within the curtain wall inspection area were extracted from the fused point cloud obtained in Section 4.2. Although the overall facade in this area is curved, the neighborhood of an individual SSAN is relatively small, allowing the local substrate surface to be approximated as a plane. Statistical outlier removal was applied using 20 nearest neighbors and a standard-deviation multiplier of 1.5. Local background planes were fitted using RANSAC with a distance threshold of 3 mm. A protrusion-distance threshold of 35 mm was used to retain candidate protruding structures, and individual SSAN clusters were obtained through spatial clustering with a tolerance of 35 mm.

**Figure 22:**
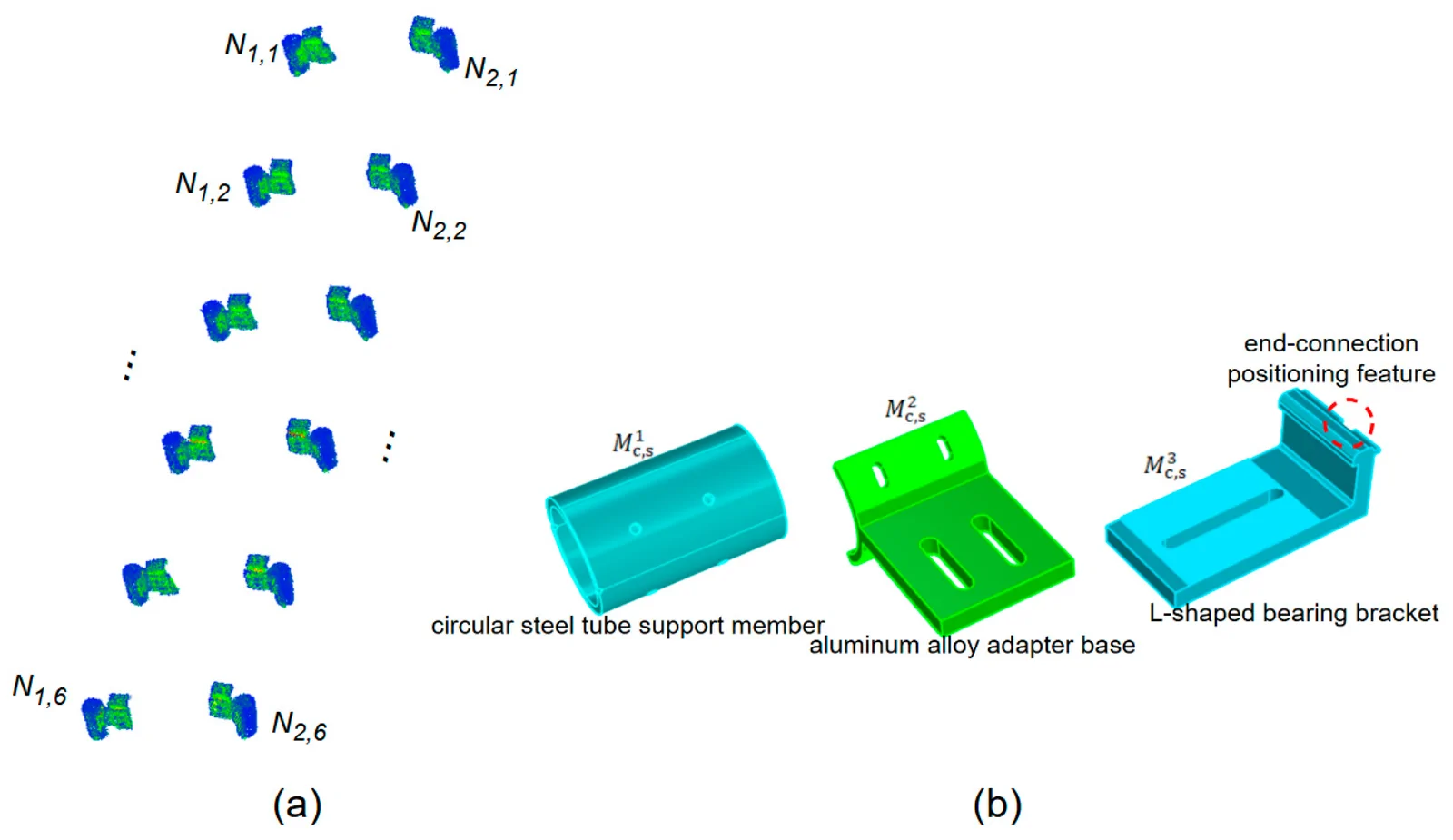
Indexing and component composition of SSANs: (**a**) SSAN indexing; (**b**) component composition.

The SSANs in the experimental area were logically indexed using the spatial indexing method established in Section 3.2.2. The experimental area contained three arrangement strips of curtain wall units, each comprising 16 curtain wall units. According to the SSAN configuration in this case, the circular steel tube support member, aluminum alloy adapter base, and L-shaped bearing bracket were denoted by $M_{c,s}^{1}$, $M_{c,s}^{2}$, and $M_{c,s}^{3}$, respectively. The indices (*c*,*s*) were inherited from SSAN *N_c_*_,*s*_, where *c* identifies the control group and *s* identifies the node serial number within that group. Superscripts 1, 2, and 3 identify the internal component types. The limiting slot of the L-shaped bearing bracket was defined as the end-connection positioning feature for subsequent multi-point attachment geometric compatibility assessment. The SSAN indices and internal component composition are shown in Figure 22.

#### 4.3.2. Extraction and Installation Checking of Circular Steel Tube Support Members

The circular steel tube support member $M_{c,s}^{1}$ is the primary support reference component of the SSAN. Its position and layout affect the installation references of the aluminum alloy adapter base and L-shaped bearing bracket. In this case, the center of the circular tube cross-section was defined as representative point $P_{c,s}^{1}$. Based on the tube diameter of 83 mm, the local cross-sectional direction was determined from the observable geometry at the tube end. The design radius was then used as a geometric constraint to separate the tube surface points from the SSAN point-cloud cluster and fit the circular cross-section, with a fitting distance threshold of 4 mm.

After the circular tube cross-sectional centers were obtained, *d*(*P*,*P*) was used to represent the distances between adjacent representative points within the same control group. These distances describe the spacing relationships among SSANs within each group. The representative points in each control group were then projected onto the local curtain wall reference plane, and their layout direction was fitted using the least-squares method. The fitted measured line was defined as the layout baseline $L_{c}^{1}$ of the circular steel tube support members. The point-to-line deviation *d*(*P*,*L*) describes the deviation from the layout baseline within each group. The included angle between the layout baselines of different groups and the distances between corresponding representative points describe the relative layout relationship $r_{L}\left(L,L\right)$ between SSAN groups.

**Figure 23:**
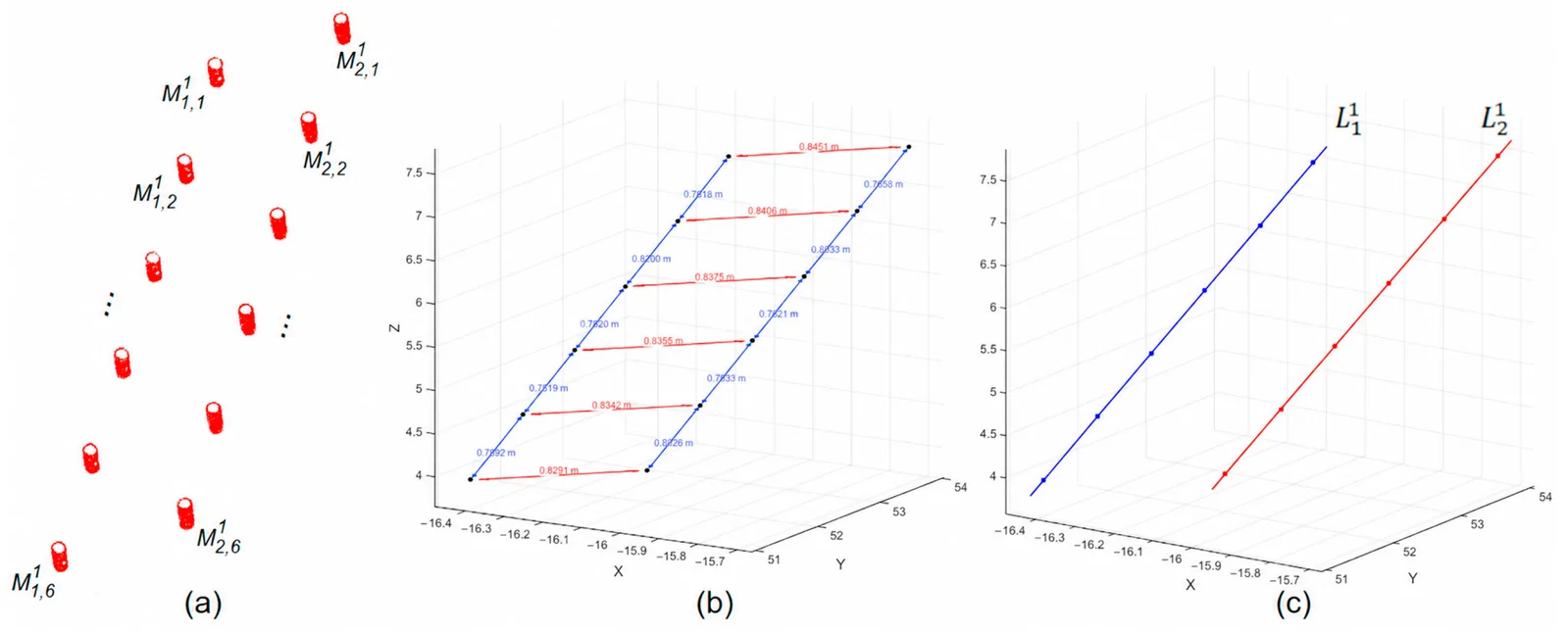
Point–line checking of circular steel tube support members: (**a**) extracted members; (**b**) representative-point distances; (**c**) fitted layout baselines.

Based on the cylindrical geometry of the circular steel tube support members and their role as the primary support reference within the SSANs, representative-point distances, point-to-line deviations, and layout-baseline relationships were selected to characterize their installation states. These indicators describe SSAN spacing, within-group layout alignment, and between-group layout. The extraction and point–line checking results are shown in Figure 23. Table 5 and Table 6 jointly constitute the component-level measured checking results $R^{1}$ of the circular steel tube support members. Table 5 presents the representative-point distances *d*(*P*,*P*), whereas Table 6 presents the point-to-line deviations *d*(*P*,*L*) and layout-baseline relationships $r_{L}\left(L,L\right)$.

As shown in Table 5, the distances between adjacent representative points and those between representative points with the same serial number in different control groups describe the measured layout dimensions of the circular steel tube support members. Table 6 shows that the RMS deviations of the representative points from the two layout baselines were 4.19 mm and 1.60 mm, respectively, and the included angle between the fitted lines was 0.034°. These results indicate stable layout alignment of the circular steel tube support members within the two control groups. Together, these results constitute the component-level measured checking results $R^{1}$ and provide the basis for subsequent end-feature analysis and tracing of out-of-range SSANs.

#### 4.3.3. Extraction and Installation Checking of Aluminum Alloy Adapter Bases

The aluminum alloy adapter base $M_{c,s}^{2}$ is located between the circular steel tube support member and the L-shaped bearing bracket. Its installation state affects the bearing direction and limiting relationship of the end-connection component. In this case, the installation reference surface of the aluminum alloy adapter base was defined as feature plane $F_{c,s}^{2}$, and the geometric center of the feature plane was defined as representative point $P_{c,s}^{2}$.

The point clouds of the aluminum alloy adapter bases were extracted from the SSAN point-cloud clusters, and their primary installation reference surfaces were identified using a RANSAC distance threshold of 2 mm and a clustering tolerance of 10 mm. After plane fitting, the normal vector of each feature plane was extracted. The angles between the normal vectors of adjacent or corresponding feature planes were calculated to characterize the plane-to-plane relationship $r_{F}\left(F,F\right)$, thereby describing the relative installation orientations of the aluminum alloy adapter bases. The distances *d*(*P*,*P*) between representative points were also calculated to characterize their relative positional states.

**Figure 24:**
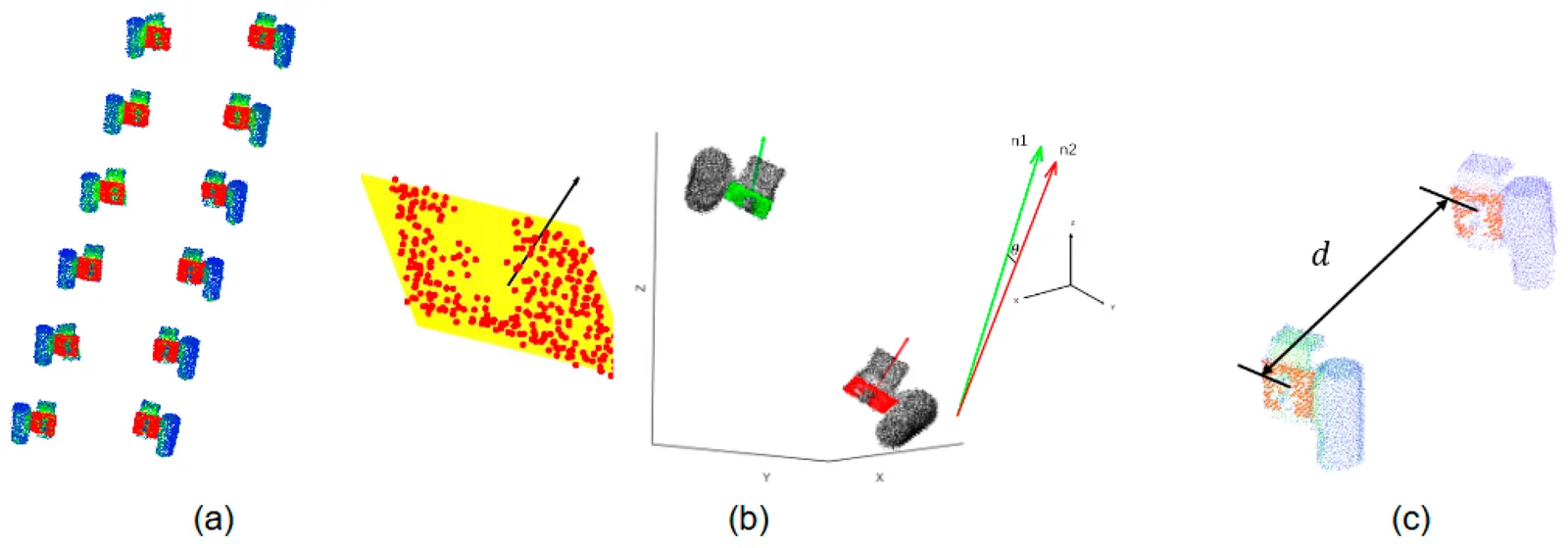
Feature-plane checking of aluminum alloy adapter bases: (**a**) extracted feature planes; (**b**) feature-plane normals; (**c**) representative-point distances.

Based on the planar geometry of the aluminum alloy adapter bases and their role in supporting and positioning the L-shaped bearing brackets, feature-plane orientation relationships and representative-point distances were selected to characterize their installation states. The feature-plane extraction and checking results are shown in Figure 24. Table 7 constitutes the component-level measured checking results $R^{2}$ of the aluminum alloy adapter bases, including the calculated *d*(*P*,*P*) and $r_{F}\left(F,F\right)$ values.

As shown in Table 7, the angles between the feature-plane normals of the aluminum alloy adapter bases were generally small, and no abrupt orientation changes were observed. This indicates good orientation consistency among the installation reference surfaces. The distances between adjacent representative points within each group reflect local installation differences along the layout direction. The distances between representative points with the same serial number in different control groups describe the transverse relative positions between the SSAN groups. Together, these results constitute the component-level measured checking results $R^{2}$ and provide an intermediate-component basis for tracing the sources of deviations in the end-connection positioning features of the L-shaped bearing brackets.

#### 4.3.4. Extraction of End-Connection Positioning Features of L-Shaped Bearing Brackets

The L-shaped bearing bracket $M_{c,s}^{3}$ is the end-connection component that directly engages with the unit-side hanger. It is installed on aluminum alloy adapter base $M_{c,s}^{2}$ and can be adjusted along the plane of the adapter base plate. In this case, the midpoint of the limiting slot in the L-shaped bearing bracket was defined as end-connection positioning feature point $P_{c,s}^{3}$. Because the slot is relatively small, its boundary may be discontinuous or locally missing in the point cloud. Therefore, its position was indirectly extracted using the upper feature plane, outer boundary, and fixed structural dimensions between the slot and the component boundary.

**Figure 25:**
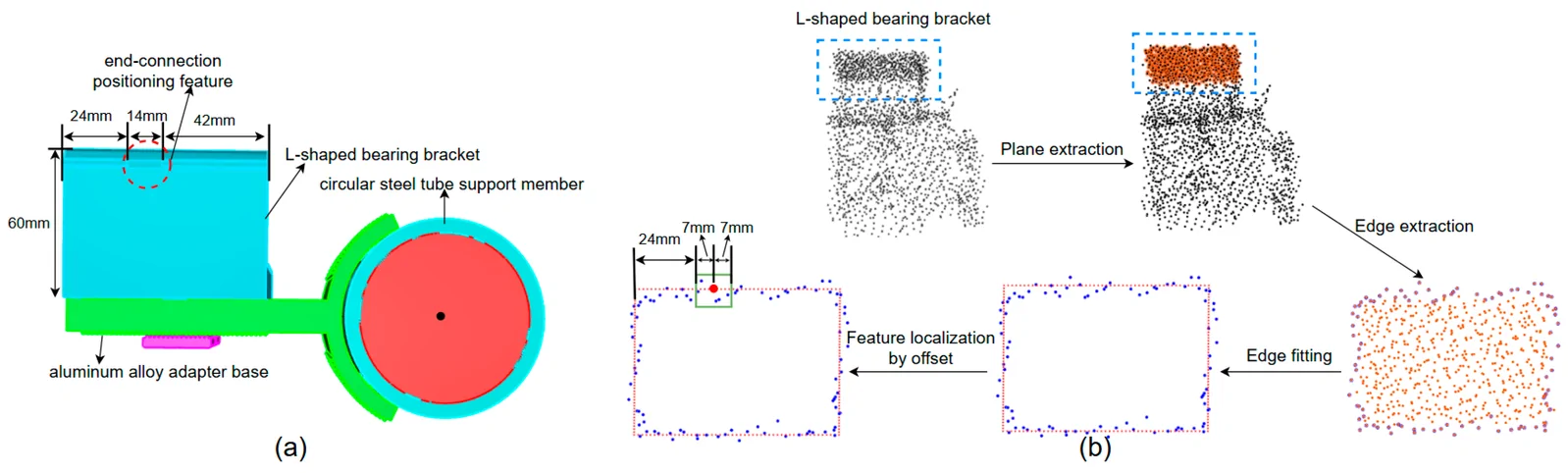
Extraction of positioning features of L-shaped bearing brackets: (**a**) feature location; (**b**) extraction result.

As shown in Figure 25, the upper feature plane of the L-shaped bearing bracket was first extracted, and its boundary was identified and fitted. The design dimensions in Figure 25 give a slot-midpoint offset of 31 mm from the left reference boundary, comprising the 24 mm edge offset and half of the 14 mm slot width. This offset was applied along the fitted upper boundary to estimate the slot-midpoint position. The resulting feature point provides a geometric reference for checking and adjusting the end position of the L-shaped bearing bracket.

**Figure 26:**
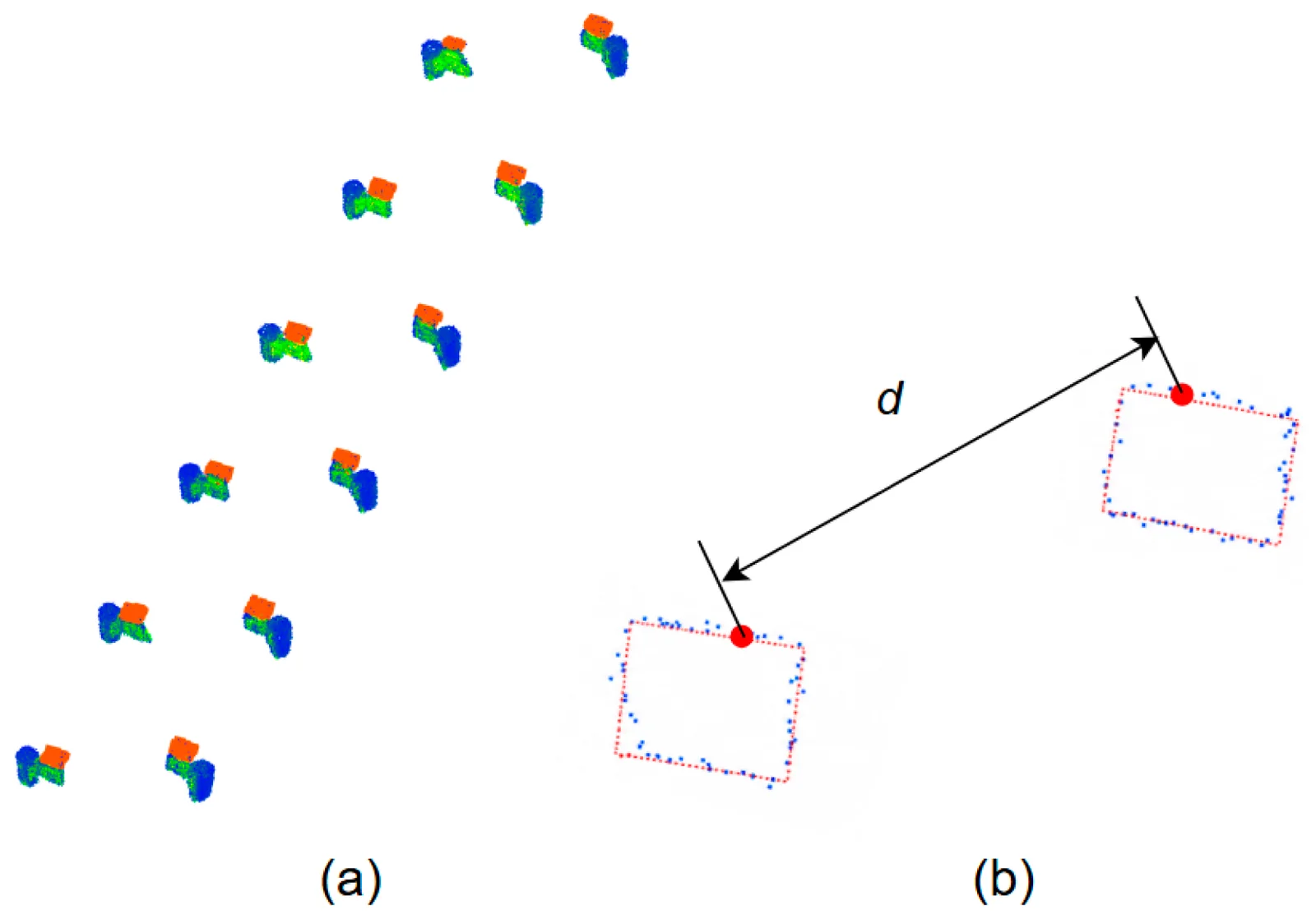
Distance calculation of positioning features of L-shaped bearing brackets: (**a**) extracted feature points; (**b**) adjacent feature-point distance.

After the end-connection positioning feature points were obtained for all SSANs, *d*(*P*,*P*) was used to calculate the distances between adjacent feature points within the same control group and between feature points with the same serial number in different control groups. The feature-point extraction and distance calculation results are shown in Figure 26. Table 8 constitutes the component-level measured checking results $R^{3}$ of the L-shaped bearing brackets.

As shown in Table 8, the distances between the end-connection positioning feature points directly represent the measured attachment dimensions of the corresponding SSAN groups. The distances between adjacent feature points within each control group describe within-group attachment-point spacing, whereas the distances between feature points with the same serial number in different control groups describe transverse attachment relationships between the SSAN groups. Together, these results constitute the component-level measured checking results $R^{3}$ and directly provide the basis for determining unit-side hanger target layout distances, identifying out-of-range relationships, and tracing associated SSANs in Section 4.4. Because $M_{c,s}^{3}$ can be adjusted along the plane of the aluminum alloy adapter base plate, $R^{3}$ can also be used to identify the L-shaped bearing brackets that require adjustment during support-side rechecking.

Because the limiting-slot midpoint was indirectly derived from the upper feature plane, fitted component boundary, and predefined structural offset, its positioning accuracy is influenced by several sources, including point-cloud measurement error, multi-source registration, geometric fitting, and the structural offset used for indirect positioning. The manufacturer-specified nominal range accuracy of the Trimble X7 used in this study is 2 mm, while the quantitative TLS–UAV registration evaluation in Section 4.2.2 yielded a point-to-plane RMSE of 3.100 mm.

### 4.4. Curtain Wall Unit Installation Checking and Hanger Pre-Adjustment Decision Output

Based on the component-level measured checking results of SSANs obtained in Section 4.3, the measured relationships among end-connection positioning features were converted into target layout parameters for unit-side hangers, and installation feasibility was evaluated. To illustrate the calculation process, two adjacent curtain wall units, *U*_1,1_ and *U*_1,2_, were selected from the experimental area.

#### 4.4.1. Establishment of Attachment Correspondences

**Figure 27:**
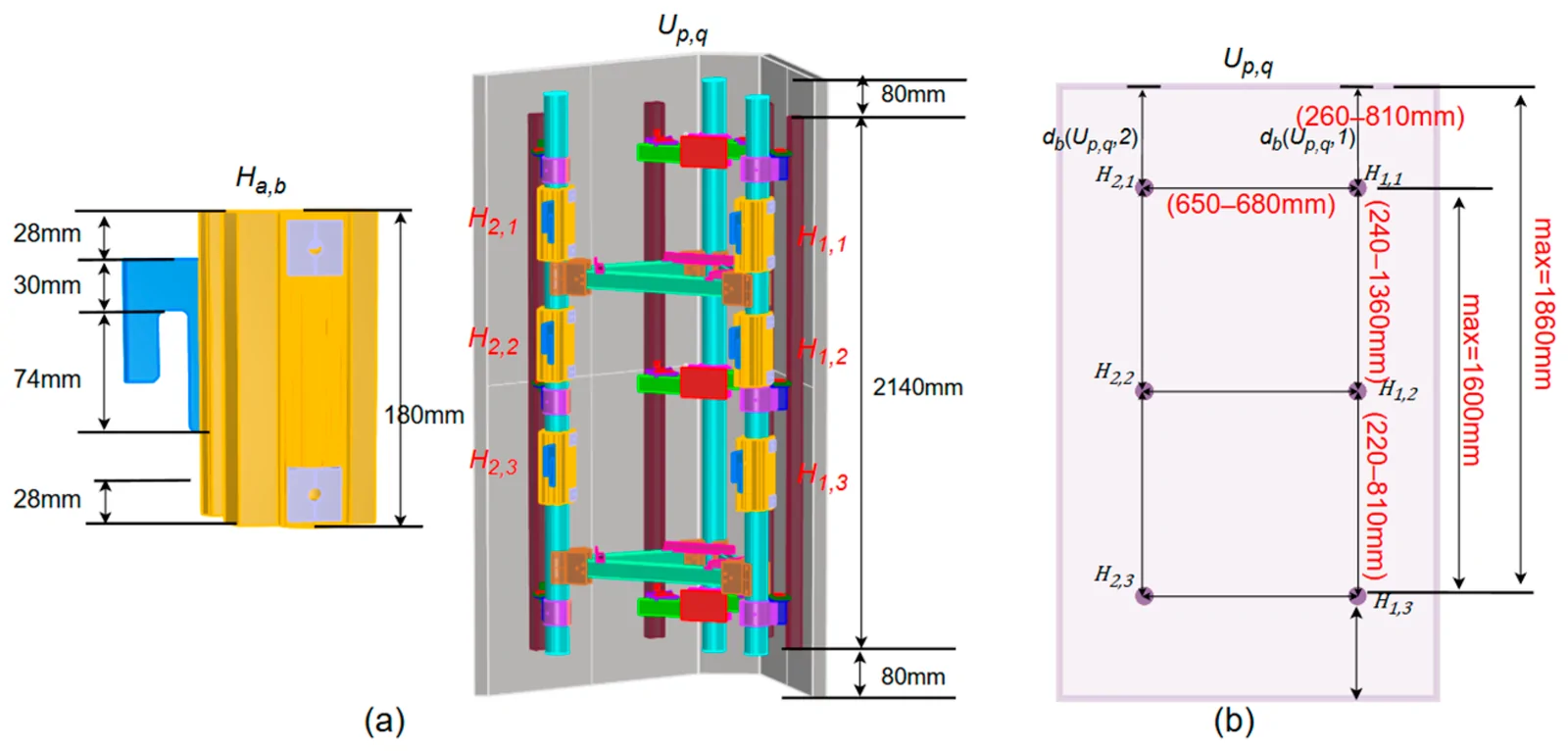
Unit-side hanger layout and adjustable range: (**a**) hanger configuration; (**b**) hanger layout and adjustable range.

Each curtain wall unit *U_p_*_,*q*_ in this case contains six unit-side hangers arranged in two local control groups, with three serially numbered hangers in each group. In the local indexing scheme, *a* = 1,2 denotes the local control-group index, whereas *b* = 1,2,3 denotes the hanger serial number within that group. To maintain consistency with the physical left-to-right and top-to-bottom hanger arrangement shown in Figure 27a, the unit-side hanger array *H_p_*_,*q*_ is displayed as:

$$H_{p,q}=[\begin{array}{cc} H_{1,1} & H_{2,1}\\ H_{1,2} & H_{2,2}\\ H_{1,3} & H_{2,3} \end{array}]$$

This display form follows the physical arrangement of the hangers. The engineering layout remains defined as two local control groups, each containing three serially numbered hangers.

The adjustment range of the unit-side hangers is jointly determined by the curtain wall unit dimensions, hanger adjustment travel, and edge installation clearance, as shown in Figure 27b. This range provides the basis for determining whether measured support-side attachment relationships can be accommodated through hanger pre-adjustment.

According to the SSAN indices in the experimental area, the SSAN group *Ω_p_*_,*q*_ corresponding to curtain wall unit *U_p_*_,*q*_ is displayed in the same physical order as:

$$\Omega_{p,q}=[\begin{array}{cc} N_{2p-1,3q-2} & N_{2p,3q-2}\\ N_{2p-1,3q-1} & N_{2p,3q-1}\\ N_{2p-1,3q} & N_{2p,3q} \end{array}]$$

Accordingly, unit-side hanger *H_a_*_,*b*_ is paired with the SSAN at the same local index (*a*,*b*). In the subsequent calculations, end-connection positioning feature point $P_{c,s}^{3}$ of SSAN *N_c_*_,*s*_ is used to determine the target layout relationships of the corresponding unit-side hanger.

#### 4.4.2. Multi-Point Attachment Compatibility Assessment Results

Based on the attachment correspondences established in Section 4.4.1, the measured distances between the end-connection positioning feature points reported in Table 8 were converted into the corresponding target layout distances of the unit-side hangers. Distances between adjacent feature points within the same local control group were mapped to the corresponding within-group hanger distances, whereas distances between feature points with the same serial number in different local control groups were mapped to the corresponding transverse hanger distances.

For curtain wall unit *U*_1,1_, the nominal comparison shows that one transverse target hanger distance is slightly greater than the upper adjustment limit shown in Figure 27b:

(15) $$d_{P_{1,1}^{3},P_{2,1}^{3}}=682.9\;\mathrm{mm}>d_{H_{1,1},H_{2,1}}^{\max}=680\;\mathrm{mm}$$

The calculated value exceeds the upper adjustment limit by 2.9 mm. Given the measurement and feature-positioning uncertainties discussed in Section 4.3.4, the corresponding SSANs *N*_1,1_ and *N*_2,1_ are flagged for field rechecking to determine whether support-side adjustment is required.

By contrast, all target hanger layout distances corresponding to curtain wall unit *U*_1,2_ fall within the allowable adjustment ranges and can be accommodated through unit-side hanger pre-adjustment.

#### 4.4.3. Boundary Positioning and Joint-Width Calculation Results

After the internal multi-point attachment geometric compatibility of each unit was assessed, the positional relationship between the unit-side hanger array and the unit design control edges was further checked. The case facade is a curved and inclined region. Therefore, the design control edges corresponding to different local control groups are not located at the same elevation and must be determined separately according to the local position of each end-connection positioning feature.

For curtain wall unit *U*_1,1_, the design elevation of the upper unit boundary corresponding to SSAN *N*_1,1_ is 71.167 m, whereas the measured elevation of its end-connection positioning feature point is 70.864 m. Their difference is:

*d_b_*(*U*_1,1_,1) = 71.167 − 70.864 = 0.303 m = 303 mm (16)

Therefore, the boundary positioning distance of local control group 1 in *U*_1,1_ is 303 mm. This value falls within the allowable boundary layout range shown in Figure 27b, indicating that the positional relationship between the hanger array and the unit design control edge satisfies the layout requirement. The boundary positioning distance *d_b_*(*U*_1,1_,2) for the other local control group in *U*_1,1_ was calculated using the same method.

For the adjacent curtain wall unit *U*_1,2_, the boundary positioning distances were calculated using the measured coordinates of the corresponding end-connection positioning feature points, the curtain wall unit height of 2300 mm, and the design joint width of 20 mm. This calculation yielded *d_b_*(*U*_1,2_,1) and *d_b_*(*U*_1,2_,2) for the two local control groups. These boundary positioning distances, together with the internal target hanger layout distances, were used as inputs for the subsequent pre-adjustment decision output.

#### 4.4.4. Pre-Adjustment Decision Output

The results of the multi-point attachment geometric compatibility assessment in Section 4.4.2 and the overall pose and joint-width coordination calculation in Section 4.4.3 were combined to convert the measured relationships among SSAN end-connection positioning features into target unit-side hanger layout parameters and installation decisions. The outputs include target hanger distances, boundary positioning distances, associated SSANs requiring rechecking or adjustment, and installation decisions, as listed in Table 9.

For curtain wall unit *U*_1,1_, both boundary positioning distances fall within the allowable ranges shown in Figure 27. Therefore, overall pose and joint-width coordination do not constitute a limiting condition. However, the measured transverse target hanger distance is 682.9 mm, nominally exceeding the maximum unit-side hanger adjustment range of 680 mm by 2.9 mm. The associated SSANs are therefore recorded as nodes requiring rechecking before installation. If field rechecking confirms that the relationship exceeds the allowable range, the corresponding support-side component adjustment can then be performed according to the traced component-level checking results in Section 4.3.

For curtain wall unit *U*_1,2_, all calculated target hanger layout distances and boundary positioning distances fall within the adjustment or allowable ranges. These relationships can therefore be accommodated through unit-side hanger pre-adjustment before lifting.

In terms of computational efficiency, the automated processing of the investigated dataset required approximately 2.0 min on the workstation specified in Table 1. This included approximately 0.3 min for TLS–UAV registration and fusion and 1.7 min for SSAN extraction, geometric-feature processing, component-level checking, and hanger pre-adjustment decision generation. Field data acquisition and photogrammetric model reconstruction were not included in this computation time.

### 4.5. Curtain Wall Installation Performance Validation

**Figure 28:**
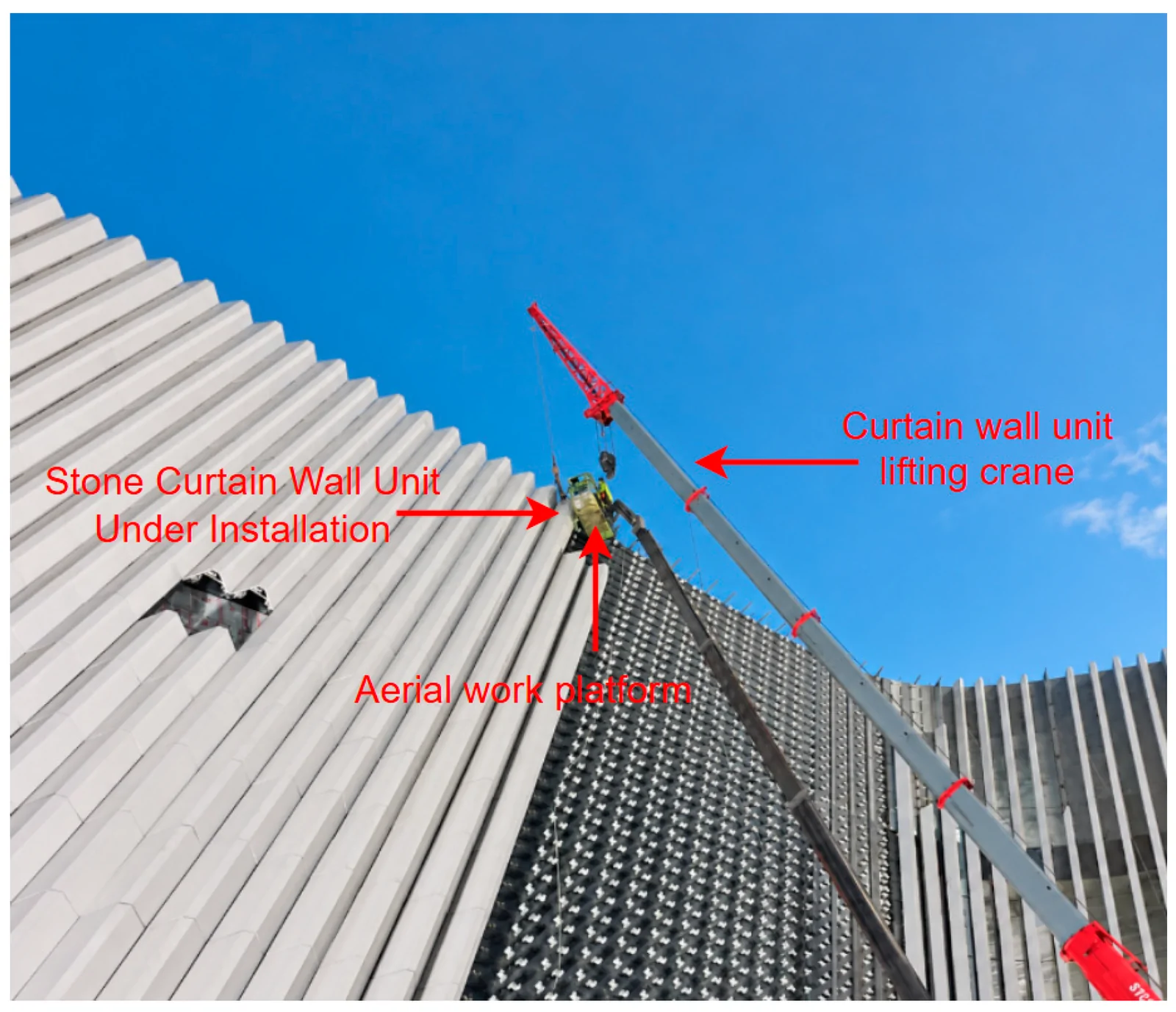
Field installation of curtain wall units during the validation process.

An on-site comparative installation validation was conducted on three curtain wall strips, each comprising 16 curtain wall units, for a total of 48 units. Two strips (32 units) were installed using the traditional manual method, while one strip (16 units) was installed using the proposed method. The installation sequence followed the actual on-site construction schedule and was not randomized. The same construction crew, which had prior experience in conventional curtain wall installation, and the same lifting equipment were used for both methods under the same project-site conditions. The validation considered the installation time per curtain wall unit, the number of on-site hanger adjustments, and post-installation joint-width uniformity. The field installation process during the validation is illustrated in Figure 28.

#### 4.5.1. Installation Efficiency Comparison

During traditional manual lifting, unit-side hangers are repeatedly adjusted according to their on-site engagement conditions. Because curtain wall units are relatively large and the hangers are located behind the units, adjustment at height is difficult and can increase lifting delays and site coordination requirements. With the proposed method, hanger pre-adjustment and installation decisions are completed before lifting based on the measured states of the SSANs. On-site lifting therefore mainly involves attachment, rechecking, and fastening.

The installation efficiency comparison is presented in Table 10. The reported durations represent the average time required per curtain wall unit for each installation stage. For the traditional method, the average total installation time was 70 min per unit, whereas the proposed method required 48 min per unit. The lifting and attachment stage decreased from 50 min to 20 min per unit. The average number of on-site adjustments per curtain wall unit also decreased from 15 to 4.

#### 4.5.2. Installation Quality Validation

Post-installation joint width was used as the installation quality indicator. The traditional and proposed installation groups were evaluated using the same field measurement procedure at the same facade elevation, with a design joint width of 20 mm. A total of 32 curtain wall units in the traditional group and 16 units in the proposed group were evaluated. The mean joint width reported for each group represents the average of the joint-width values obtained from the evaluated curtain wall units. The measured range, maximum deviation, and variation range were also calculated from these joint-width records, as summarized in Table 11.

As shown in Table 11, the measured joint-width range for the traditional method was 16.8–27.4 mm, with a variation range of 10.6 mm, whereas that for the proposed method was 18.6–23.1 mm, with a variation range of 4.5 mm. The mean joint widths of both groups were close to the design value of 20 mm. For the investigated installation area, the narrower range obtained with the proposed method suggests improved joint-width consistency.

## 5. Conclusions

This study addressed the pre-lifting geometric compatibility of multi-point attachment unit systems in dry-hanging stone curtain walls and proposed an SSAN installation-checking and hanger pre-adjustment method based on UAV photogrammetry and TLS point clouds. Through multi-source point cloud fusion, the method captures the measured geometric states of installed SSANs, establishes SSAN indices and component-level geometric checking relationships, extracts end-connection positioning features, and converts the measured relative relationships within SSAN groups into unit-side hanger pre-adjustment parameters, SSAN rechecking indices, and installation decisions.

The engineering case study demonstrated that the proposed method can characterize the measured geometric relationships of SSANs and their internal components and assess the geometric compatibility between end-connection positioning features and unit-side hanger arrays. By linking out-of-range relationships to the corresponding SSANs and component-level checking results, the method provides construction personnel with explicit hanger pre-adjustment parameters and SSAN indices for rechecking before lifting. The on-site comparative installation results showed that the average total installation time per curtain wall unit decreased from 70 min to 48 min, the lifting and attachment time decreased from 50 min to 20 min, and the average number of on-site adjustments per curtain wall unit decreased from 15 to 4. For the installation area with a design joint width of 20 mm, the post-installation joint-width range narrowed from 16.8–27.4 mm to 18.6–23.1 mm.

Several limitations remain. First, the field evaluation was conducted as an engineering case study on three installation strips within a single project, without randomized allocation. Installation sequence, strip-specific conditions, and crew experience may have influenced the observed differences. The results were summarized using group-level statistics, while inferential statistical analysis and detailed evaluation of unit-to-unit variability were not included. Further validation across additional projects with larger datasets is needed to assess the robustness of the observed differences.

Second, the method depends on high-quality point cloud data. For inspection areas at high elevations, across large spans, or under severe occlusion, high-accuracy point cloud acquisition equipment and stable data-processing conditions are required. The current geometric validation combined check-target comparisons at different elevations with registration-residual analysis over the overlapping geometries of 138 SSAN components. Future studies will incorporate independent measurements of representative end-connection positioning features and quantitative uncertainty evaluation to further assess feature-level accuracy and decision reliability near the adjustment limits.

Third, the extraction of end-connection positioning features relies on observable component surfaces and local structural dimensions. For different SSAN configurations, the spatial indexing method and Point–Line–Plane representation can be retained, while the component-specific feature definitions, geometric constraints, and tolerance models need to be adapted according to the actual connection geometry and point-cloud observability. Edge- and image-assisted feature extraction, template-based matching, and local surface reconstruction may be further investigated to improve feature identification under occlusion and incomplete sampling and to extend the framework to larger curved and free-form curtain wall geometries.

Fourth, this study primarily focused on pre-lifting geometric compatibility and hanger pre-adjustment and did not integrate structural load-bearing capacity, connection forces, or long-term deformation into a unified assessment framework. Future research may map the generated hanger pre-adjustment parameters to the coordinate system and motion constraints of automated pre-adjustment equipment and integrate construction-process monitoring and structural-performance checking into a more comprehensive digital construction-control workflow for multi-point attachment installation in dry-hanging stone curtain walls.

## Figures and Tables

**Table 1:** Configuration of experimental equipment and software.

Equipment/Software	Specification
DJI M300 RTK	RTK-enabled UAV platform; Compatible with multiple gimbal camera platforms
DJI Zenmuse P1	Full-frame photogrammetry gimbal camera; Sensor: 35.9 × 24 mm CMOS; Pixel size: 4.4 μm; Standard lens: 35 mm F2.8; FOV: 63.5°
Trimble X7 3D Laser Scanner	Terrestrial laser scanner; Field of view: 360° × 282°; Scan time: as fast as 2 min 34 s (with image capture); Scan rate: up to 500 kHz; Range: 0.6–80 m; Range accuracy: 2 mm; Angular accuracy: 21”; Laser angular resolution: 0.0011 (2 min), 0.0005 (4 min), 0.0004 (7 min)
DJI Terra	3D reconstruction
CloudCompare	Open-source point cloud processing software
CPU	Intel Xeon Silver 4210R
GPU	NVIDIA GeForce RTX 3090 24 GB
RAM	128 GB

**Table 2:** Comparison of target-based geometric measurements between the TLS and UAV photogrammetric models at different elevations.

Floor	TLS Target Spacing (mm)	UAV Target Spacing (mm)	Difference (mm)	Mean |ΔX| (mm)	Mean |ΔY| (mm)	Mean |ΔZ| (mm)
Level 1	3482	3478	4	1.7	1.7	1.2
Level 2	3479	3482	3	1.6	1.9	1.5
Level 3	3483	3482	1	1.8	2.1	1.6
Level 4	3480	3479	1	1.7	2.0	1.8
Level 5	3482	3479	3	2.0	2.3	1.9

**Table 3:** Quantitative evaluation of TLS–UAV registration over the 138 evaluated SSAN components.

Indicator	Result
Number of evaluated SSAN components	138
FPFH candidate correspondences	11,920
RANSAC inlier correspondences	72
Valid point pairs for residual evaluation	219,323
Point-to-point RMSE	5.564 mm
Point-to-plane RMSE	3.100 mm
Point-to-plane median	2.369 mm
Point-to-plane P95	6.081 mm

**Table 4:** Comparison of relative geometric coverage among TLS-only, UAV-only, and fused TLS–UAV point clouds.

Dataset	Relative Geometric Coverage (%)	Difference Relative to TLS-Only (Percentage Points)
TLS only	68.3	-
UAV only	95.2	26.9
TLS–UAV fusion	100.0	31.7

**Table 5:** Checking results of representative-point distances of circular steel tube support members.

Component Index *i*	Control Group *c*	Serial Number *s*	$d\left(P_{c,s}^{i},P_{c\prime ,s\prime }^{i}\right)$/mm
$M_{c,s}^{1}$	1	(1, 2)	781.8
		(2, 3)	800.0
		(3, 4)	782.3
		(4, 5)	781.9
		(5, 6)	799.2
	2	(1, 2)	783.6
		(2, 3)	803.3
		(3, 4)	782.1
		(4, 5)	783.3
		(5, 6)	802.6
	(1, 2)	1	845.1
		2	840.6
		3	837.6
		4	835.5
		5	834.2
		6	829.1

**Table 6:** Checking results of layout baseline deviation of circular steel tube support members.

Component Index *i*	Control Group *c*	Serial Number *s*	$d\left(P_{c,s}^{1},L_{c}^{1}\right)$/mm	$L_{c}^{1}$’s RMS/mm	$r_{L}\left(L_{c}^{1},L_{c\prime }^{1}\right)$/°
$M_{c,s}^{1}$	1	1	6.89	4.19	0.034
		2	6.40		
		3	1.85		
		4	1.18		
		5	0.82		
		6	3.36		
	2	1	0.03	1.60	
		2	1.77		
		3	0.99		
		4	1.07		
		5	2.12		
		6	2.38		

**Table 7:** Checking results of aluminum alloy adapter bases.

Component Index *i*	Control Group *c*	Serial Number *s*	$r_{F}\left(F_{c,s}^{i},F_{c\prime ,s\prime }^{i}\right)$/°	$d\left(P_{c,s}^{i},P_{c\prime ,s\prime }^{i}\right)$/mm
$M_{c,s}^{2}$	1	(1, 2)	2.20	736.5
		(2, 3)	2.55	764.5
		(3, 4)	1.08	817.6
		(4, 5)	1.07	756.2
		(5, 6)	1.00	762.7
	2	(1, 2)	3.04	773.9
		(2, 3)	1.08	762.5
		(3, 4)	1.13	796.8
		(4, 5)	0.72	762.3
		(5, 6)	2.59	761.3
	(1, 2)	1	2.88	670.7
		2	2.19	675.4
		3	1.17	683.6
		4	2.13	683.5
		5	1.89	675.2
		6	3.06	692.1

**Table 8:** Checking results of end-connection positioning feature distances of L-shaped bearing brackets.

Component Index *i*	Control Group *c*	Serial Number *s*	$d\left(P_{c,s}^{i},P_{c\prime ,s\prime }^{i}\right)$/mm
$M_{c,s}^{3}$	1	(1, 2)	743.1
		(2, 3)	767.2
		(3, 4)	813.5
		(4, 5)	754.6
		(5, 6)	761.2
	2	(1, 2)	770.9
		(2, 3)	758.7
		(3, 4)	800.4
		(4, 5)	774.8
		(5, 6)	762.6
	(1, 2)	1	682.9
		2	657.0
		3	660.7
		4	663.6
		5	673.7
		6	665.2

**Table 9:** Hanger pre-adjustment and installation decision output for curtain wall units.

Curtain Wall Unit *U_p_*_,*q*_	Installation Decision	Target Relation	Target Value/mm	Associated Nodes *N_c_*_,*s*_
*U*_1,1_	Rechecking required	*d_b_*(*U*_1,1,1)	303	
		*d*(*H*_1,1_, *H*_1,2_)	743.1	
		*d*(*H*_1,2_, *H*_1,3_)	767.2	
		*d_b_*(*U*_1,1_,2)	265.3	
		*d*(*H*_2,1_, *H*_2,2_)	754.6	
		*d*(*H*_2,2_, *H*_2,3_)	761.2	
		*d*(*H*_1,1_, *H*_2,1_)	682.9	*N*_1,1_, *N*_2,1_
		*d*(*H*_1,2_, *H*_2,2_)	657.0	
		*d*(*H*_1,3_, *H*_2,3_)	660.7	
*U*_1,2_	Install after pre-adjustment	*d_b_*(*U*_1,2_, 1)	306.8	
		*d*(*H*_1,1_, *H*_1,2_)	770.9	
		*d*(*H*_1,2_, *H*_1,3_)	758.7	
		*d_b_*(*U*_1,2_, 2)	274.9	
		*d*(*H*_2,1_, *H*_2,2_)	774.8	
		*d*(*H*_2,2_, *H*_2,3_)	762.6	
		*d*(*H*_1,1_, *H*_2,1_)	663.6	
		*d*(*H*_1,2_, *H*_2,2_)	673.7	
		*d*(*H*_1,3_, *H*_2,3_)	665.2	

**Table 10:** Comparison of average installation time and number of adjustments per curtain wall unit.

	Curtain Wall Units, *n*	Hanger Pre-Adjustment/min	Pre-Lifting Rigging/min	Lifting and Attachment/min	Bolt Fastening/min	Total Time/min	Number of Adjustments
Traditional	32	0	5	50	15	70	15
Proposed method	16	8	5	20	15	48	4

**Table 11:** Joint width comparison.

	Curtain Wall Units	Design Joint Width/mm	Measured Range/mm	Mean Joint Width/mm	Maximum Deviation/mm	Variation Range/mm
Traditional	32	20	16.8–27.4	21.6	7.4	10.6
Proposed method	16	20	18.6–23.1	20.8	3.1	4.5

## Data Availability

Data will be made available on request.
